# ALDH1A1 drives prostate cancer metastases and radioresistance by interplay with AR- and RAR-dependent transcription

**DOI:** 10.7150/thno.88057

**Published:** 2024-01-01

**Authors:** Ielizaveta Gorodetska, Anne Offermann, Jakob Püschel, Vasyl Lukiyanchuk, Diana Gaete, Anastasia Kurzyukova, Vera Freytag, Marie-Therese Haider, Christina S Fjeldbo, Simona Di Gaetano, Franziska Maria Schwarz, Shivaprasad Patil, Angelika Borkowetz, Holger H. H. Erb, Aria Baniahmad, Jovan Mircetic, Heidi Lyng, Steffen Löck, Annett Linge, Tobias Lange, Franziska Knopf, Ben Wielockx, Mechthild Krause, Sven Perner, Anna Dubrovska

**Affiliations:** 1 OncoRay-National Center for Radiation Research in Oncology, Faculty of Medicine and University Hospital Carl Gustav Carus, Technische Universität Dresden and Helmholtz-Zentrum Dresden-Rossendorf, Dresden, Germany.; 2 Institute of Pathology, University Hospital Schleswig-Holstein, Luebeck, Germany; Pathology, Research Center Borstel, Leibniz Lung Center, Borstel, Germany.; 3 Institute of Clinical Chemistry and Laboratory Medicine, Technische Universität Dresden, Dresden, Germany.; 4 Technische Universität Dresden, CRTD - Center for Regenerative Therapies TU Dresden and Center for Healthy Aging, Faculty of Medicine and University Hospital Carl Gustav Carus, Technische Universität Dresden, Dresden, Germany.; 5 Institute of Anatomy and Experimental Morphology, Center for Experimental Medicine, University Cancer Center Hamburg, University Medical Center Hamburg-Eppendorf, Germany.; 6 Department of Radiation Biology, Oslo University Hospital, Oslo, Norway.; 7 Helmholtz-Zentrum Dresden-Rossendorf, Institute of Radiooncology-OncoRay, Dresden, Germany.; 8 German Cancer Consortium (DKTK), partner site Dresden and German Cancer Research Center (DKFZ), Heidelberg, Germany.; 9 Department of Urology, University Hospital and Faculty of Medicine, Technische Universität Dresden, Dresden, Germany.; 10 Institute of Human Genetics, Jena University Hospital, Friedrich Schiller University, Jena, Germany.; 11 Department of Physics, University of Oslo, Oslo, Norway.; 12 National Center for Tumor Diseases (NCT), partner site Dresden: German Cancer Research Center (DKFZ), Heidelberg; Faculty of Medicine and University Hospital Carl Gustav Carus, Technische Universität Dresden, and Helmholtz-Zentrum Dresden-Rossendorf (HZDR), Dresden, Germany.; 13 Department of Radiotherapy and Radiation Oncology, Faculty of Medicine and University Hospital Carl Gustav Carus, Technische Universität Dresden, Dresden, Germany.; 14 Institute of Anatomy I, Cancer Center Central Germany, Jena, University Hospital, Jena, Germany.

**Keywords:** prostate cancer, bone metastases, cancer stem cells, aldehyde dehydrogenase, RARA, androgen receptor, retinoic acid

## Abstract

**Rationale:** Current therapies for metastatic osseous disease frequently fail to provide a durable treatment response. To date, there are only limited therapeutic options for metastatic prostate cancer, the mechanisms that drive the survival of metastasis-initiating cells are poorly characterized, and reliable prognostic markers are missing. A high aldehyde dehydrogenase (ALDH) activity has been long considered a marker of cancer stem cells (CSC). Our study characterized a differential role of ALDH1A1 and ALDH1A3 genes as regulators of prostate cancer progression and metastatic growth.

**Methods:** By genetic silencing of ALDH1A1 and ALDH1A3 *in vitro*, in xenografted zebrafish and murine models, and by comparative immunohistochemical analyses of benign, primary tumor, and metastatic specimens from patients with prostate cancer, we demonstrated that ALDH1A1 and ALDH1A3 maintain the CSC phenotype and radioresistance and regulate bone metastasis-initiating cells. We have validated ALDH1A1 and ALDH1A3 as potential biomarkers of clinical outcomes in the independent cohorts of patients with PCa. Furthermore, by RNAseq, chromatin immunoprecipitation (ChIP), and biostatistics analyses, we suggested the molecular mechanisms explaining the role of ALDH1A1 in PCa progression.

**Results:** We found that aldehyde dehydrogenase protein ALDH1A1 positively regulates tumor cell survival in circulation, extravasation, and metastatic dissemination, whereas ALDH1A3 plays the opposite role. ALDH1A1 and ALDH1A3 are differentially expressed in metastatic tumors of patients with prostate cancer, and their expression levels oppositely correlate with clinical outcomes. Prostate cancer progression is associated with the increasing interplay of ALDH1A1 with androgen receptor (AR) and retinoid receptor (RAR) transcriptional programs. Polo-like kinase 3 (PLK3) was identified as a transcriptional target oppositely regulated by ALDH1A1 and ALDH1A3 genes in RAR and AR-dependent manner. PLK3 contributes to the control of prostate cancer cell proliferation, migration, DNA repair, and radioresistance. ALDH1A1 gain in prostate cancer bone metastases is associated with high PLK3 expression.

**Conclusion:** This report provides the first evidence that ALDH1A1 and PLK3 could serve as biomarkers to predict metastatic dissemination and radiotherapy resistance in patients with prostate cancer and could be potential therapeutic targets to eliminate metastasis-initiating and radioresistant tumor cell populations.

## Introduction

Prostate cancer (PCa) is the second most commonly diagnosed malignancy in men, accounting for 1.4 million new cases worldwide in 2020 1. Fortunately, PCa can often be diagnosed at the early locoregional stages by testing the prostate-specific antigen (PSA) levels and in most cases can be cured by surgery or radiotherapy with or without androgen deprivation. Nevertheless, 15% of PCa patients are diagnosed with advanced disease and have an increased risk of developing a metastatic state with a five-year survival rate below 30% 2. The disseminated PCa cells have a high tropism to the bone, and most patients with advanced PCa develop bone metastases 3. Current therapies for metastatic osseous disease, including radiotherapy and systemic treatment, frequently fail to provide a durable treatment response by preventing metastatic growth. PCa is an androgen-driven malignancy. Androgen deprivation therapy (ADT) is the standard of care for patients with PCa at advanced stages of the disease, either as combined treatment with surgery or radiotherapy or, in palliative situations, as the sole long-treatment modality. However, with a long time of androgen deprivation, the disease progresses to castration-resistant prostate cancer (CRPC). Metastatic CRPC is associated with an unfavourable prognosis and a mean survival time of about 16-18 months 4. The treatment response of metastatic PCa is highly heterogeneous. To date, there are only limited therapeutic options for metastatic PCa, the mechanisms that drive the survival of metastasis-initiating cells (MIC) are poorly characterized, and reliable prognostic markers are missing.

While the tumor-initiating properties of cancer cells are plastic and reversible, the populations of cancer stem cells (CSCs) capable of initiating and maintaining tumor growth and relapse is of utmost clinical importance 5. PCa progression is associated with tumor dedifferentiation and gain of CSC features 6. The fundamental properties of CSCs, including self-renewal and differentiation potential, make them a unit of tumor evolution and a critical target for anti-cancer treatment 7. Our own and other previous studies suggested several intrinsic and extrinsic mechanisms that confer CSC radioresistance through upregulating DNA repair, activation of the cell survival pathways, and lowering oxidative stress 8-11. Furthermore, CSCs drive metastatic tumor growth. Metastasis-initiating cells (MICs) are CSC subpopulations that exert their tumor-initiating properties in adverse microenvironments. As for now, the role of distinct CSC subpopulations as prognostic indicators in patients with PCa remains uncertain, and prostate MICs are not yet characterized.

We have previously described aldehyde dehydrogenase (ALDH) activity as one of the markers of PCa stem cells 10. ALDH is an essential class of nicotinamide adenine dinucleotide phosphate (NAD(P)^+^)-dependent enzymes protecting cells against oxidative stress by oxidizing endogenous and exogenous aldehydes to their corresponding carboxylic acids 12. The ALDH1A1 and ALDH1A3 proteins have been described as the dominant isoforms responsible for ALDH activity in PCa cells 13. Both ALDH isoforms synthesize retinoic acid (RA) from retinol. The primary mediators of RA signaling are ligand-activated transcriptional factors, the retinoic acid receptors (RAR), and the retinoid X receptors (RXR). RAR and RXR form homo- or heterodimers and bind to retinoic acid‐responsive elements in the regulatory sequences of target genes. RAR and RXR interplay with androgen receptor (AR)-driven transcription program and may function as either AR repressors or coactivators depending on the target gene and bound ligand 14.

Our study investigated the cellular processes and molecular mechanisms regulated by ALDH proteins that contribute to the maintenance of PCa metastasis-initiating and radioresistant cells. By genetic silencing of *ALDH1A1* and *ALDH1A3 in vitro* in xenografted zebrafish and murine models, and by comparative immunohistochemical analyses of benign, primary tumor, and metastatic specimens from patients with PCa, we demonstrated that *ALDH1A1* and *ALDH1A3* maintain the CSC phenotype and radioresistance and regulate bone metastasis-initiating cells. We have validated ALDH1A1 and ALDH1A3 as potential biomarkers of clinical outcomes in the independent cohorts of patients with PCa. Furthermore, by RNAseq, chromatin immunoprecipitation (ChIP) and biostatistics analyses, we suggested the molecular mechanisms explaining the role of ALDH1A1 in PCa progression. For the first time, we demonstrated that ALDH1A1 and ALDH1A3 play an opposite role in the regulation of PCa metastasis, and this function is mediated by their interplay with AR through regulation of the RAR-dependent transcriptional targets.

## Results

### ALDH1A1 and ALDH1A3 regulate the CSC phenotype and PCa radiosensitivity

**Figure 1:**
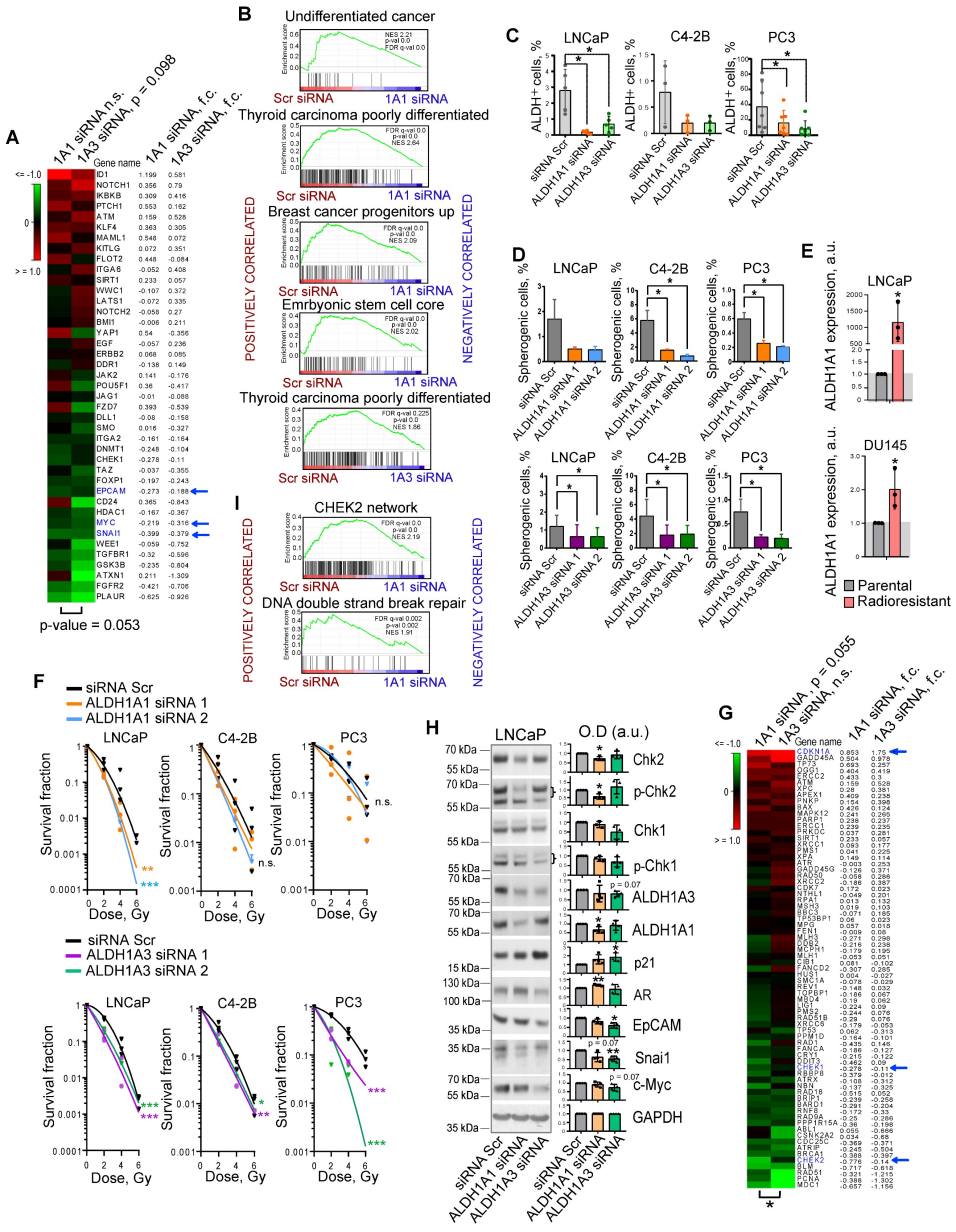
** ALDH1A1 and ALDH1A3 regulate the CSC phenotype and PCa radiosensitivity.** (**A**) The RNA sequencing analysis of LNCaP cells transfected with ALDH1A1 siRNA, ALDH1A3 siRNA, or scrambled siRNA revealed that ALDH1A3 downregulation is associated with a decrease in CSC-related gene expression at a larger extent than ALDH1A1 (n = 41, RT2 Cancer Stem Cells geneset). Blue arrows indicate genes whose expression levels were confirmed by western blotting in panel H. (**B**) Gene Set Enrichment Analysis (GSEA) for genes significantly up- or down regulated upon ALDH1A1 or ALDH1A3 knockdown revealed that deregulated genes are associated with stemness and differentiation. (**C**) Flow cytometry analysis of ALDH^+^ population upon ALDH1A1 and ALDH1A3 knockdown shows decreased Aldefluor enzymatic activity. N≥3; Error bars = SD; *p < 0.05. (**D**) Percentage of the spherogenic cells after ALDH1A1 or ALDH1A3 depletion. The bar graph represents the % of spherogenic cells upon ALDH1A1 and ALDH1A3 knockdown. N ≥ 3; Error bars = SD; *p < 0.05. (**E**) Quantitative real-time PCR (RT-qPCR) analysis of ALDH1A1 expression in LNCaP and DU145 parental and radioresistant cell lines. N = 3; Error bars = SD; *p < 0.05. (**F**) Relative cell radiosensitivity was analyzed by 2D radiobiological colony forming assay after siRNA-mediated knockdown of ALDH1A1 or ALDH1A3 in LNCaP, C4-2B, or PC3 cells. Cells transfected with scrambled (Scr) siRNA were used as control. N ≥ 3; Error bars = SD; *p < 0.05; **p < 0.01; ***p < 0.001. (**G**) The RNA sequencing analysis of LNCaP cells transfected with ALDH1A1 siRNA, ALDH1A3 siRNA or scrambled siRNA revealed that ALDH1A1 downregulation is associated with a decrease in DNA damage response (DDR) and repair genes (n = 71, RT2 DNA Damage Signaling Pathway geneset); *p < 0.05. Blue arrows indicate genes whose expression levels were confirmed by western blotting in panel H. (**H**) Western blot analysis of selected genes from the datasets in Figure 1A and Figure 1G. Representative images of one of four independent repeats are shown; Error bars = SD; *p < 0.05; **p < 0.01. (**I**) GSEA analysis for genes significantly up- or down regulated upon ALDH1A1 knockdown revealed their association with Chk2 signaling and DNA double strand break repair.

We and others have shown that PCa cells with high ALDH activity (ALDH^+^) defined by Aldefluor analysis are enriched for CSCs, have a high activation of β-catenin/WNT signaling pathway, and increased migratory properties 10, 11, 15. We showed that ALDH^+^ cells have relatively high radioresistance and more efficiently resolve DNA double-strand breaks induced by irradiation than ALDH^-^ cells 10, 11. Previous studies identified nine members of the ALDH family contributing to Aldefluor activity 16. However, only the ALDH1A1 isoform was correlated with Aldefluor activity in the PCa patient's tissue specimens 13. Analysis of gene expression profiling of ALDH^+^ and ALDH^-^ cell populations isolated by FACS from DU145 PCa cells revealed that only *ALDH1A3* was significantly upregulated in the ALDH^+^ population (Figure S1A). Also, *ALDH1A3* showed a high correlation with the fraction of ALDH^+^ cells in four PCa cell lines (r = 0.980) (Figure S1B). siRNA-mediated knockdown of both *ALDH1A1* and *ALDH1A3* induced deregulation of genes involved in CSC maintenance, although *ALDH1A3* has a higher impact on the regulation of the CSC gene set (Figure 1A). Gene Set Enrichment Analysis (GSEA) 17 confirmed that genes downregulated after the knockdown of both ALDH1A1 and ALDH1A3 were enriched in the datasets associated with normal stem cells, tumor progenitors and poorly differentiated cancer (Figure 1B). Analysis of the ALDH enzymatic activity in PCa cells after *ALDH1A1* and *ALDH1A3* knockdown revealed a more than 3-fold decrease in the ALDH^+^ population and therefore confirmed the role of both genes in its regulation (Figure 1C). Since ALDH^+^ cells exhibit stem-like properties, we next analyzed an association of *ALDH1A1* and *ALDH1A3* with CSC phenotype under serum-free sphere-forming conditions. Cells with genetically silenced *ALDH1A1* and *ALDH1A3* expression showed significantly decreased sphere number and size (Figure 1D, Figure S1C,D). One of the common features of PCa stem cells is their relative resistance to conventional therapies such as radiation therapy 18. Our previous studies demonstrated that radioresistant (RR) PCa cells possess an enhanced expression of cancer stem cell markers, including high ALDH activity and activated WNT/β-catenin signaling pathway 10, 11. We have analyzed the expression of nine ALDH isoforms responsible for Aldefluor activity in two PCa models with acquired radioresistance and found that only *ALDH1A1* was highly upregulated in both RR cell lines (Figure 1E, Figure S1E). Radiobiological clonogenic analyses demonstrated that the knockdown of both genes results in PCa cell radiosensitization (Figure 1F, Figure S1F,G). Similar results were obtained after PCa cell pretreatment with high concentrations (≥ 10^-5^ M) of all-trans retinoic acid (ATRA), which inhibits ALDH activity 19 and expression of ALDH1A1 and ALDH1A3 genes (Figure S1H,I,J). siRNA-mediated knockdown of ALDH1A1 and ALDH1A3 deregulates similarly the expression of some genes involved in DNA damage response (DDR) and repair, such as *CHEK1* (Chk1), *CHEK2* (Chk2), and *CDKN1A* (p21). However, *ALDH1A1* has a significantly higher impact on the regulation of the DDR and DNA repair gene set (Figure 1G,H). GSEA analysis confirmed that genes deregulated by ALDH1A1 knockdown are associated with CHEK2 signaling network and DNA double-strand break repair (Figure 1I). Furthermore, the knockdown of *ALDH1A1* upregulates *AR*, a transcriptional regulator of DNA repair genes in PCa^20^, suggesting a balancing feedback mechanism (Figure 1H). These experiments suggest ALDH1A1 and ALDH1A3 as regulators of a transcriptional program driving CSC phenotype and radioresistance in prostate cancer cells.

### Expression levels of ALDH1A1 and ALDH1A3 genes are mutually regulated

**Figure 2:**
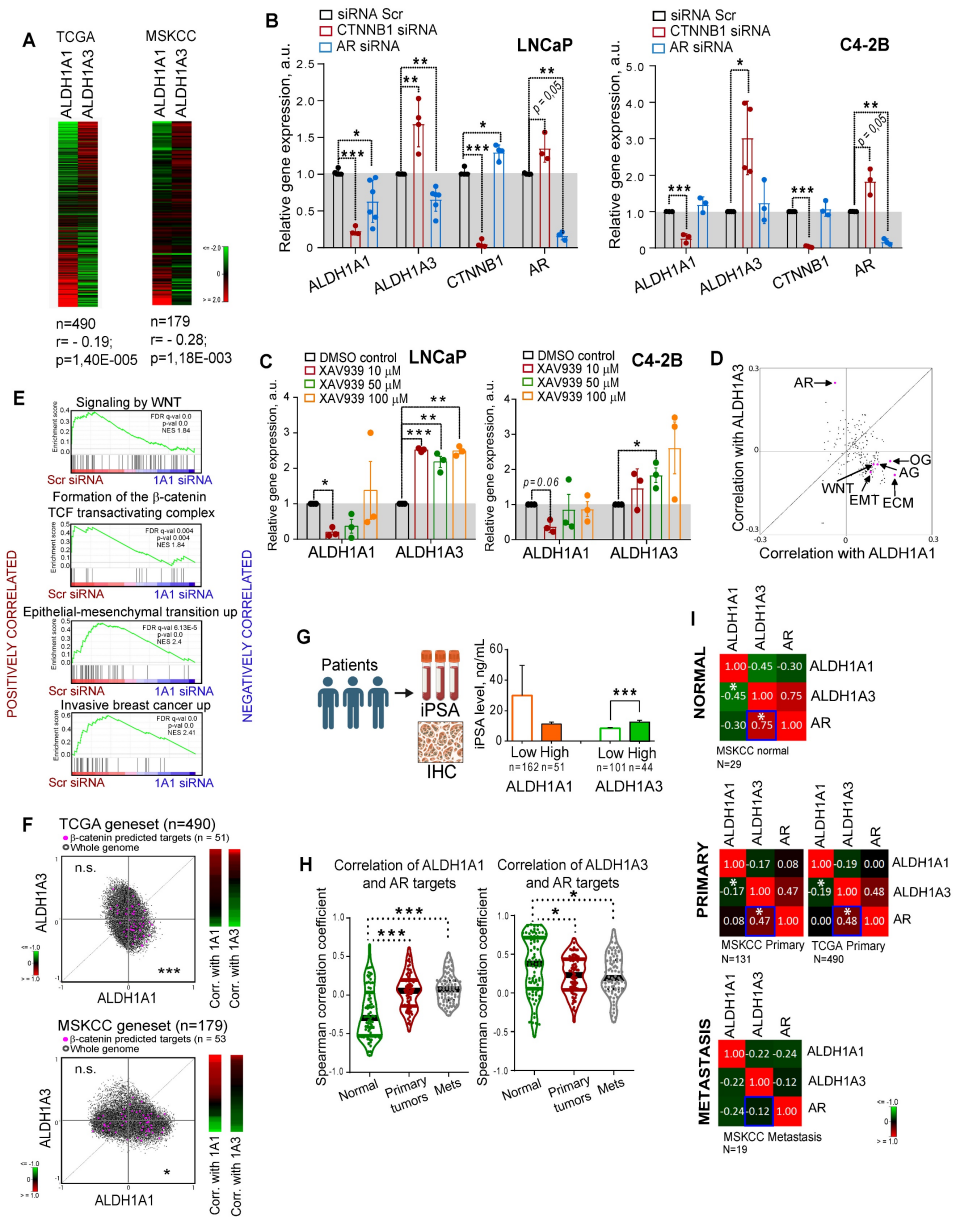
** Expression levels of ALDH1A1 and ALDH1A3 genes are mutually regulated.** (**A**) Analysis of the TCGA dataset for patients with PCa (n = 490) and MSKCC cohort (n = 179, including normal tissue samples, n = 29; primary prostate cancer samples, n = 131; and metastatic prostate cancer samples, n = 19) showed a weak negative correlation of ALDH1A1 and ALDH1A3 genes. (**B**) Relative mRNA expression of ALDH1A1, ALDH1A3, CTNNB1, and AR upon the knockdown of CTNNB1 and AR genes. N ≥ 3; Error bars = SD. *p < 0.05; **p < 0.01; ***p < 0.001. (**C**) Analysis of ALDH1A1 and ALDH1A3 genes expression upon inhibition of WNT signaling pathway with XAV939 inhibitor. DMSO-treated cells were used as control. The cells were serum-starved in RPMI medium with 3% FBS for 24 h, followed by treatment with XAV939 at different concentrations. N = 3; Error bars = SEM. *p < 0.05; **p < 0.01; ***p < 0.001. (**D**) The correlation of the common molecular pathways with ALDH1A1 and ALDH1A3 in a provisional prostate cancer TCGA dataset (n = 490). AR: androgen receptor signaling targets; WNT: WNT signaling targets; ECM: extracellular matrix and adhesion molecules; EMT: epithelial to mesenchymal transition; AG: angiogenesis; OG: osteogenesis. n = 84 for all genesets; Gene lists are provided in Table S4. (**E**) GSEA analysis for genes significantly deregulated upon ALDH1A1 knockdown revealed their association with WNT/β-catenin inhibition, EMT and tumor invasion. (**F**) Correlation of mRNA expression for β-catenin predicted targets 27 with ALDH1A1 and ALDH1A3 in the TCGA and MSKCC patient cohorts. ***p < 0.001; *p < 0.05; n.s.- non-significant. (**G**) Correlation of the initial preoperative prostate-specific antigen (iPSA) serum level in patients with PCa with protein expression of ALDH1A1 and ALDH1A3 in tumor tissues (Lübeck cohort). Error bars = SEM. ***p < 0.001. (**H**) Correlation of ALDH1A1 and ALDH1A3 expression levels with the expression of the AR transcriptional targets in normal tissues (MSKCC dataset, n = 29), primary tumors (MSKCC dataset, n = 131), and metastatic tumors (MSKCC dataset, n = 19). The gene list for AR transcriptional targets is provided in Table S4. Statistical analysis was performed by Kruskal-Wallis rank sum test for multiple independent samples. Conover p-values were further adjusted by Benjamini-Hochberg FDR method; *p < 0.05; ***p < 0.001. (**I**) Correlation of ALDH1A1, ALDH1A3, and AR expression levels in normal tissues (MSKCC dataset, n = 29), primary tumors (MSKCC dataset, n = 131; TCGA dataset, n = 490), and metastatic tumors (MSKCC dataset, n = 19); *p < 0.05. Blue squares show the declining correlation of ALDH1A3 and AR expression from normal tissues through primary tumor to metastases.

Both ALDH1A1 and ALDH1A3 proteins possess a similar physiological role in the biosynthesis of retinoic acid 12, 21, and both appeared as regulators of CSC properties and radioresistance in our study. Therefore, we next analyzed whether the expression of these genes is mutually exclusive, which could be an indirect confirmation of their functional redundancy. Analyses of the gene expression data using the publicly available PCa dataset (PRAD) from The Cancer Genome Atlas (TCGA) (n = 490) 22 that almost exclusively includes primary tumors as well as MSKCC dataset (n = 179) 23 that includes normal tissues, primary and metastatic tumors revealed a weak but significant negative correlation between these genes (*r =* -0.195 for TCGA and *r =* -0.28 for MSKCC) (Figure 2A). Next, we assumed that transcriptional compensation might occur if these genes have similar functions in PCa models. The relationship between *ALDH1A1* and *ALDH1A3* was analysed in knockdown experiments. Considering that two siRNAs used for previous experiments showed similar trends, we used the pooled siRNA for further investigations. We found that genetic silencing of *ALDH1A3* induces downregulation of *ALDH1A1*; however, the depletion of *ALDH1A1* significantly increased *ALDH1A3* mRNA expression in all analysed cell lines (Figure S2A). Analysis of the other members of the ALDH family contributing to Aldefluor activity showed that only ALDH1A2 was highly upregulated after ALDH1A3 knockdown (Figure S2B).

Discordance was also observed by analysis of the ALDH1A1 and ALDH1A3 expression in response to the knockdown of key PCa drivers playing a role at the initial stage of tumor development (AR) or in the advanced PCa (β-catenin) 24, 25. ALDH1A1 was downregulated after β-catenin knockdown in all tested cell models: androgen-sensitive cells derived from a metastatic lymph node lesion (LNCaP); their derivative cell line C4-2B which is osteotropic, AR^+^ and androgen-independent, and bone metastasis-derived AR^-^ PC3 cells (Figure 2B, Figure S2C). In contrast, ALDH1A3 and AR were upregulated after β-catenin knockdown in LNCaP and C4-2B cells. Both ALDH1A1 and ALDH1A3 expression levels were inhibited by AR knockdown in androgen-sensitive LNCaP cells, however there was no such regulation in the androgen-independent osteotropic C4-2B cells. These results were confirmed by using XAV939, a chemical inhibitor of tankyrase inducing β-catenin degradation (Figure 2C, Figure S2D), and by cell treatment with enzalutamide, an AR inhibitor confirming previous data that ALDH1A3 is a direct AR transcriptional target 26 (Figure S2E).

To further explore the role of ALDH genes in PCa, we used TCGA gene expression dataset to analyse the potential correlation of *ALDH1A1* and *ALDH1A3* with gene sets corresponding to 198 common molecular pathways. This analysis revealed a significant positive correlation of *ALDH1A1* with several gene sets related to cancer progression, e.g. WNT signaling, angiogenesis, osteogenesis, extracellular matrix and adhesion molecules. On the other hand, *ALDH1A3* was strongly associated with expression of the AR signaling targets (Figure 2D). GSEA confirmed that genes downregulated in response to the ALDH1A1 knockdown were enriched in the datasets associated with WNT/β-catenin signaling, epithelial-mesenchymal transition, and tumor invasion (Figure 2E). We have additionally verified a significant correlation of ALDH1A1 with β-catenin target genes previously described for colorectal cancer models 27 (Figure 2F). To further investigate the link between ALDH genes and AR signaling, we correlated the initial preoperative prostate-specific antigen (iPSA) serum level in patients with PCa with protein expression of ALDH1A1 and ALDH1A3 and observed a significantly increased iPSA level in patients with ALDH1A3 overexpressing tumors (Figure 2G). There was no significant difference in the nuclear AR expression between tumors with or without ALDH1A1 or ALDH1A3 expression (Figure S2F), although a transcription program driven by nuclear AR might be repressed or activated depending on the presence of many regulatory proteins 28. While *ALDH1A1* negatively correlates with AR target genes in noncancerous prostate epithelium, this mutual exclusivity reduces upon tumor development.

*ALDH1A3* shows a significantly higher correlation with both AR expression and AR-driven transcriptional program, which decreases in primary tumors versus normal tissues and even more declines in metastases (Figure 2H,I). These results confirmed that although ALDH1A1 and ALDH1A3 contribute to similar biochemical mechanisms, their expression is differently driven by two key PCa regulators, AR and β-catenin, and correlates with distinct biological pathways. Therefore, we hypothesized that ALDH1A1 and ALDH1A3 might not have complete functional redundancy and may potentially contribute to the different steps of PCa development.

### ALDH proteins differentially correlate with clinical outcome

**Figure 3:**
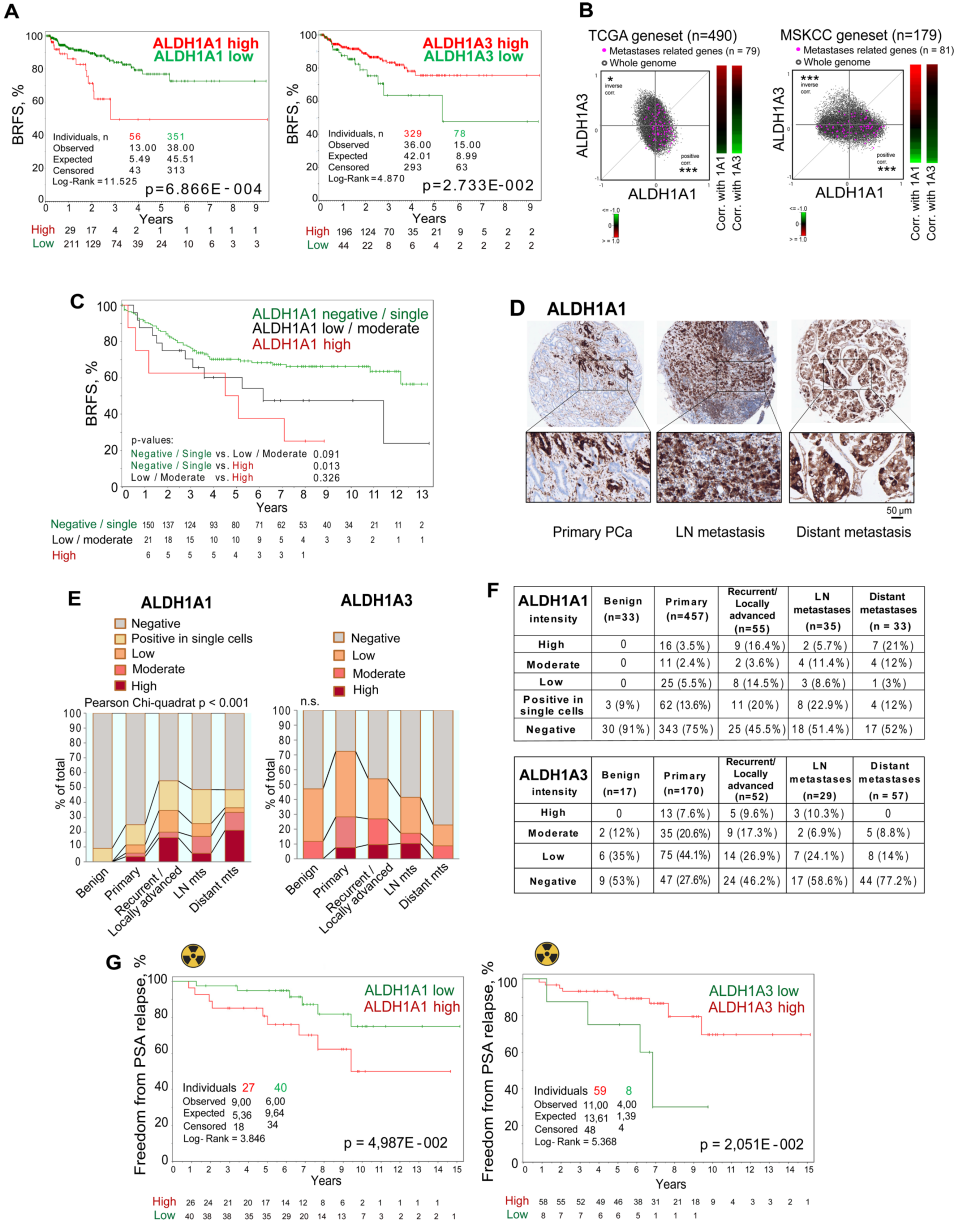
** ALDH proteins differentially regulate clinical outcome.** (**A**) The Kaplan-Meier analyses of BRFS for TCGA PRAD patients stratified by the most significant cut-off for ALDH1A1 and ALDH1A3 expression levels. (**B**) Correlation of mRNA expression for metastasis-related genes with ALDH1A1 and ALDH1A3 in the TCGA and MSKCC patient cohorts. The gene list is provided in Table S4; *p < 0.05; ***p < 0.001. (**C**) The Kaplan-Meier analysis of biochemical recurrence-free survival of patients with negative / single cells (green) compared to low/moderate (black) and high (red) ALDH1A1 expression level (Lübeck cohort). N = 205; p < 0.05. (**D**) Representative images showing ALDH1A1 expression in prostate cancer tissues at 10x and 40x magnification. ALDH1A1 is highly expressed in metastases. (**E, F**) The levels and types of ALDH1A1 (N = 613) and ALDH1A3 (N = 325) expressions in the benign prostatic hyperplasia (BPH), primary prostate cancer tissues, recurrent tumor, lymph node and distant metastasis cells (Lübeck cohort). (**G**) The Kaplan-Meier analysis of freedom from PSA relapse in patients with prostate cancer treated with radiotherapy with high (red) compared to low (green) ALDH1A1 or ALDH1A3 expression levels; N = 67 (Dresden cohort).

To investigate the predictive value of *ALDH1A1* and *ALDH1A3* gene expression, we analyzed biochemical recurrence-free survival (BRFS) of TCGA PRAD patients' cohort stratified based on the expression of those two genes. This analysis demonstrated that *ALDH1A1* and *ALDH1A3* gene expression levels oppositely correlate with clinical outcomes. While patients with high *ALDH1A3* expression exhibited better BRFS, increased expression of the *ALDH1A1* gene was associated with a worse BRFS (Figure 3A). In addition, a signature combining ALDH1A1-high with ALDH1A3-low expression has a higher correlation with BRFS than a single gene expression (Figure S3A). In further support of the opposite role of these genes in PCa progression, we found a significant positive correlation of a set of metastases-related genes and ALDH1A1, and strong anticorrelation with ALDH1A3 in the TCGA and MSKCC patient datasets (Figure 3B).

To validate the clinical relevance of our findings, we first investigated a possible association of ALDH1A1 protein expression with BRFS in a retrospective, monocentric cohort including 205 patients diagnosed with PCa (Lübeck sub-cohort with sufficient follow-up data). We found that patients with high ALDH1A1 expression in primary tumors exhibited worse BRFS compared to the negative/single-cell positive subgroup (Figure 3C). These results are consistent with the data obtained for the publicly available PRAD TCGA cohort (Figure 3A). In contrast to ALDH1A1, ALDH1A3 expression in primary tumors does not significantly correlate with patients' outcomes (Figure S3B). Analysis of the gene expression dataset from an independent cohort of patients with primary intermediate or high-risk PCa (Oslo cohort 29, n = 95) validated a negative correlation between ALDH1A1 and ALDH1A3 genes (Figure S3C). We also confirmed that ALDH1A3 negatively correlates with clinical parameters associated with cancer aggressiveness, such as pathological tumor stage, Gleason score and tumor size (Figure S3D).

Next, we performed a comparative analysis of the expression levels of ALDH1A1 (n = 613) and ALDH1A3 (n = 325) proteins in benign prostatic tissues, primary PCa tissues, tissues from locally advanced or recurrent PCa, lymph node and distant metastasis (Lübeck cohort) by immunohistochemical staining. We found that the ALDH1A1 levels in primary tumors are associated with positive lymph node (N1) status (Figure S3E). The level of ALDH1A1 increases during PCa progression. ALDH1A1 is more frequently highly expressed in distant metastases and locally advanced/recurrent tumors. In contrast, ALDH1A3 is more frequently highly expressed in primary tumor samples but not in distant metastasis (Figure 3D-F, Figure S3F). We then analyzed whether the expression of ALDH1A1 and ALDH1A3 is affected by bisphosphonates such as zolendronic acid (Zol) indicated for the treatment of bone metastases. In addition to their antiresorptive activity, bisphosphonates also demonstrated anti-cancer activity 30. Thus, we treated PCa cells with Zol and measured the mRNA expression of *ALDH1A1* and *ALDH1A3*. Expression of the *ALDH1A1* gene was inhibited in most analyzed cell lines in a dose-dependent manner. In contrast, *ALDH1A3* expression was upregulated in lymph-node metastatic cells (LNCaP) and downregulated in bone metastatic cells (PC3), suggesting that the inhibition of *ALDH1A1* could potentially contribute to the previously described anti-tumor effect of bisphosphonates 30 (Figure S3G). In addition, we analyzed androgen-responsive 22Rv1 cells transfected with the reporter plasmid where an endogenous *ALDH1A1* promoter regulates luciferase expression as we described earlier 11 (Figure S3H).

We found that Zol inhibited luciferase expression in a dose-dependent manner. However, we did not confirm this observation at the level of *ALDH1A1* mRNA expression (Figure S3G). This contradiction can be potentially explained by the presence of the constitutively active androgen receptor splice variant 7 (AR-V7) in 22Rv1 cells compared to all other used cell lines. Indeed, a previously published study suggested that AR-V7 interplays with a full-length AR in the transcription of their shared gene targets 31. Interestingly, AR is shown to be upregulated by Zol in 22Rv1 cells but not in LNCaP cells in a dose-dependent manner, suggesting that AR increase in response to the Zol treatment could play a role in the *ALDH1A1* regulation (Figure S3G). Furthermore, AR is known to negatively regulate several miRNAs including miR-29, miR-155, and miR-21, which target *ALDH1A1* gene expression 32-36. Furthermore, some *ALDH1A1*-targeted miRNAs, such as miR-29 and miR-155 are negatively regulated by BRCA1 37-39. Indeed, in our previous studies, we found that the knockdown of *BRCA1* significantly decreased the expression of ALDH1A1 and increased the expression of *ALDH1A3* mRNA in LNCaP 40. We have also observed the same mode of *ALDH1A1*, *ALDH1A3*, and *BRCA1* regulation in response to Zol treatment of LNCaP cells: Zol-induced *BRCA1* downregulation is associated with downregulation of *ALDH1A1* and upregulation of *ALDH1A3*. In contrast, *BRCA1* expression is not affected by Zol treatment in 22Rv1, and consistently, we did not observe any effect on the *ALDH1A1* and *ALDH1A3* expression in this cell line (Figure S3G). These observations suggest that the effect of Zol treatment on the *ALDH1A1* expression depends on the activation of specific oncogenic and tumor suppressor mechanisms, including AR-V7, AR and BRCA1 signaling axes.

To validate whether the expression of *ALDH1A1* and *ALDH1A3* genes also correlates with tumor radioresistance in PCa patients, we analyzed the expression of these genes in tumor tissues of patients with intermediate or high-risk localized PCa treated with radiotherapy (n = 67, Dresden cohort 9). We found a significant association of high *ALDH1A1* expression with lower relapse-free rates, whereas high expression of *ALDH1A3* is significantly associated with higher rates of freedom from PSA relapse (Figure 3G). Altogether, these findings demonstrate an opposite association of ALDH1A1 and ALDH1A3 expression with PCa clinical outcomes and their differential expression in metastatic tumors. We then hypothesized that these genes contribute differently to the regulation of PCa metastatic development.

### ALDH genes differentially regulate experimental PCa metastases

Metastatic dissemination is a multi-stage process. First, cancer cells must detach from the primary tumor, intravasate, survive in the circulation, and finally, extravasate, invade the target tissue, and colonize the metastatic site. To investigate the role of ALDH1A1 and ALDH1A3 for tumor cell survival in the bloodstream and during the extravasation process *in vivo*, we employed the larval zebrafish (*Danio rerio*) model to xenograft human prostate cells. Zebrafish represent a powerful tool for cancer research as human and zebrafish genomes share a high degree of sequence homology in 82% of disease-causing genes 41. Furthermore, the optical transparency of zebrafish larvae allows live observation of tumor cells injected into different sites 42. We used two color-coded PC3 cell lines expressing either the red fluorescent protein tdTomato or the green fluorescent protein GFP. First, we validated that these fluorescent proteins do not affect tumor cell extravasation. To do so, we co-injected PC3-GFP and PC3-tdTomato cells into the Duct of Cuvier (DoC) of Tg*(kdrl*:CFP*)* endothelial reporter transgenic zebrafish 43 at 2 days post fertilization (dpf). High-resolution imaging of the whole tail region, including the caudal hematopoietic tissue (CHT), the site of hematopoiesis at this developmental stage, was used to visualize vital and extravasated cells at 3 days post injection (dpi). Analyses of the survived cells in the bloodstream and extravasated cells in the tail region confirmed no effect of GFP or tdTomato expression on cell survival and extravasation (Figure S4A).

**Figure 4:**
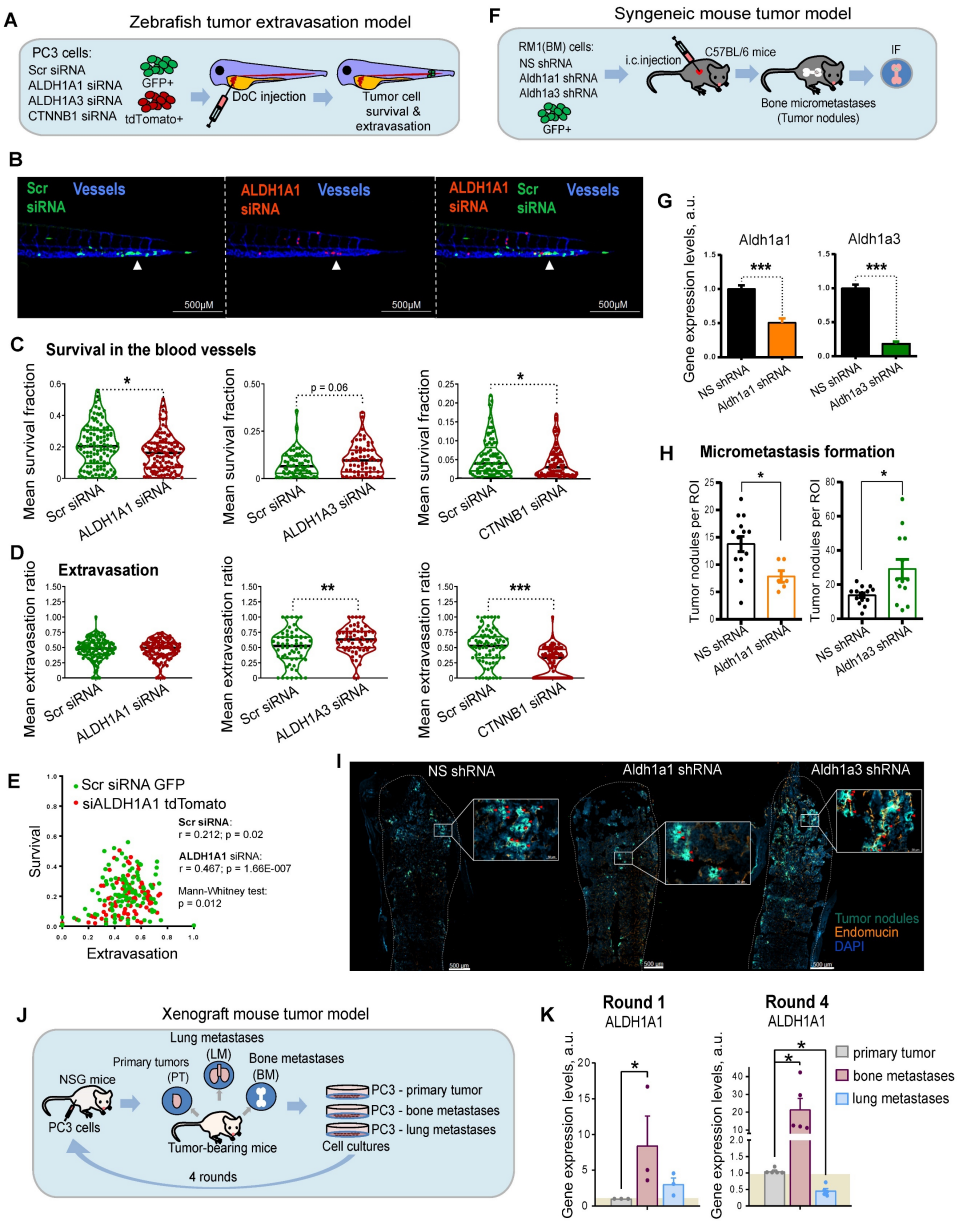
** ALDH genes differentially regulate experimental PCa metastases.** (**A**) A scheme of the experiment performed in the zebrafish tumor extravasation model. (**B**) Representative fluorescent images of the zebrafish tail. CFP (blue) - vessels; tdTomato (red) - color-coded prostate cancer PC3 cells transfected with ALDH1A1 siRNA; GFP (green) - color-coded prostate cancer PC3 cells transfected with Scr siRNA. Arrows show extravasated cells. Scale bars = 500 µm. (**C**) Quantification of survival of PC3 cells upon ALDH1A1, ALDH1A3 and CTNNB1 knockdown. ALDH1A1 N = 118, ALDH1A3 N = 64, CTNNB1 N = 85. Fish that did not have survived cells of a particular color were excluded from survival analysis; *p < 0.05. (**D**) Quantification of the extravasation potential of color-coded PC3 cells upon ALDH1A1, ALDH1A3 and CTNNB1 knockdown; **p < 0.01; ***p < 0.001. (**E**) Correlation of *in vivo* cell survival and extravasation in response to the scrambled (Scr) siRNA transfection or siALDH1A1 transfection. Dissimilarity of cell survival and extravasation after Scr siRNA or ALDH1A1 siRNA transfection was evaluated by the data dimensionality reduction followed by the Mann-Whitney U test. (**F**) A scheme of the experiment for the syngeneic mouse tumor model. Three days after intracardiac injection of mouse prostate cancer RM1(BM) GFP^+^ cells with or without Aldh1a1 and Aldh1a3 depletion, the formation of tumor nodules was detected in the bones by immunofluorescence analysis. (**G**) Validation of knockdown efficacy in the mouse prostate cancer cell line RM1(BM) transfected with shRNA against Aldh1a1 and Aldh1a3. The data are plotted relative to the control non-specific shRNA (NS) sample. Error bars = SD; ***p < 0.001. (**H**) The number of tumor nodules formed in the bone tissue upon knockdown of Aldh1a1 or Aldh1a3 in the syngeneic immunocompetent mice was analyzed by immunofluorescence (N = 4 mice/group; N of analyzed bone slides: NS shRNA = 14, Aldh1a1 shRNA = 6, Aldh1a3 shRNA = 13; ROI (region of interest) = one bone slide). Outliers were removed by the iterative Grubbs' method with α = 0.05. Statistics were performed using a two-sided Mann-Whitney U test. Error bars = SEM; *p < 0.05. (**I**) Representative immunofluorescence images of the formed tumor nodules in the Aldh1a1 shRNA, Aldh1a3 shRNA, and control NS shRNA samples. Arrows show tumor nodules. Scale bars are 500 µm and 50 µM (inserts). (**J**) A scheme of the experiment for the xenograft mouse tumor model. After subcutaneous engraftment of human prostate cancer PC3 cells, primary tumors (PT), bone marrow (BM) and lung (LM) metastasis were formed. Small pieces of surgically excised tumors were cultured *in vitro* and gave rise to sublines PC3-PT (derived from the primary tumor), PC3-BM (derived from BM metastasis), and PC3-LM (derived from lung metastasis). (**K**) qPCR analysis of ALDH1A1 expression in the PC3 cells originating from different sites: primary tumors, bone marrow metastases, and lung metastases. Cells were passaged in mice in four rounds and the sublines from the 1^st^ and 4^th^ rounds were taken for the comparative analysis. The data is plotted relative to the primary tumor samples. N ≥ 3; Error bars = SEM. *p < 0.05.

In the following experiments, PC3-tdTomato cells were used for the siRNA-mediated knockdown of *ALDH1A1, ALDH1A3*, or β-catenin (*CTNNB1*) and PC3-GFP cells transfected with scrambled siRNA (siSCR) were used as control. The pairs of siSCR/GFP and siALDH1A1/tdTomato, or siSCR/GFP and siALDH1A3/tdTomato or siSCR/GFP and siCTNNB1/tdTomato cells were co-injected into the DoC of the Tg*(kdrl*:CFP*)* zebrafish embryos at 2 dpf (Figure 4A). The survived and extravasated cells were analyzed at 3 dpi as described above. The data showed that cells depleted for *ALDH1A1* had a lower survival rate in the blood flow compared to control cells (Figure 4B,C and Figure S4B). We evaluated the extravasation potential of PC3 cells with and without *ALDH1A1* or *ALDH1A3* depletion by counting the number of extravasated cells in the tail region. Cells with suppressed *ALDH1A3* expression showed higher extravasation capacities than the siSCR control (Figure 4D). Cells with *ALDH1A1* knockdown did not show any differences in extravasation capacity. Nevertheless, we found a correlation between *in vivo* cell survival and extravasation rates in response to ALDH1A1 knockdown (*r* = 0.467, p = 1.66E-007), suggesting that ALDH1A1 is essential for the coordination of both biological processes (Figure 4E). In contrast, there was a negligible correlation between survival and extravasation in response to ALDH1A3 knockdown (Figure S4C). We also found that survival and extravasation properties define scrambled siRNA and siALDH1A1 cells as well as scrambled siRNA and siALDH1A3 cells as statistically distinct populations (p = 0.012 and p = 0.009, respectively). Moreover, we performed the same evaluation for tumor cells upon knockdown of *CTNNB1*, which has been shown to positively regulate *ALDH1A1* expression (Figure 4C,D and Figure S4B). These experiments revealed decreased survival and extravasation of cells upon *CTNNB1* depletion. However, we did not find a correlation between cell survival and extravasation that may be attributed to the low *in vivo* cell survival rate after *CTNNB1* knockdown (Figure S4D).

We then tested whether *ALDH1A1* and *ALDH1A3* regulate tumor cells homing to bone and bone marrow colonization *in vivo*. For this purpose, we employed murine RM1(BM) PCa cells with bone metastases take rate over 95% in the syngeneic immunocompetent C57BL/6 mice as discussed previously 44 (Figure 4F). We first transfected RM1(BM)-GFP cells with plasmid vectors expressing shRNA against *Aldh1a1* or *Aldh1a3* to generate stable lines with decreased target gene levels (shAldh1a1 or shAldh1a3). RM1(BM) cells transfected with a nonspecific shRNA (shNS) were used as a control. shAldh1a1 and shAldh1a3 cells showed a reduction in expression of target genes by 50% and 80% compared with shNS cells as analyzed by qPCR (Figure 4G). Next, we examined the potency of the shAldh1a1 or shAldh1a3 knockdown cells to metastasize to the bones after being injected into the left ventricles of male C57BL/6 mice. The animals were sacrificed three days post intracardiac injections, and the hind limbs (femurs and tibiae) were isolated. The homing of RM1(BM) cells to the bones and their growth therein were monitored by immunofluorescence microscopy analysis of GFP-positive tumor nodule formation in bone marrow tissue (Figure 4H,I). Bone marrow endothelium was stained with an anti-endomucin antibody. The metastatic potential of cells upon shAldh1a1 or shAldh1a3 knockdown conditions, as well as nonspecific control cells, was evaluated by the number of metastatic tumor nodules. This experiment showed that cells depleted for *Aldh1a1* formed a lower number of tumor nodules when compared to the control. At the same time, *Aldh1a3* knockdown cells exhibited a higher number of formed tumor nodules.

To further investigate the role of ALDH genes in metastasis, we measured the expression of *ALDH1A1* and *ALDH1A3* genes in the PC3-derived cell lines originating from different metastatic sites 45. For this analysis, PC3 cells were first subcutaneously injected into immunodeficient NSG mice, and small pieces of surgically excised xenograft primary tumors (PT), as well as spontaneous lung (L) and bone marrow (BM) metastases were used for *in vitro* propagation of sublines PC3-PT, PC3-L, and PC-BM, respectively (Figure 4J) 9, 45, 46. These sublines were then re-injected subcutaneously into the secondary recipient NSD mice, and the entire procedure was repeated four times. Our analysis revealed an overexpression of the *ALDH1A1* gene in the tumor bone metastatic cells compared to cells derived from primary tumors. Furthermore, this difference was substantially higher in the 4^th^ compared to the 1^st^ generation of the bone metastasis-derived cells (*8.3 f.c*. vs. *21.5 f.c*.). These results suggest a role of *ALDH1A1* in the longitudinal evolution of tumor bone metastatic properties. At the same time, the expression of *ALDH1A3* was not significantly altered in the 1^st^ and in the 4^th^ generation of the metastasis-derived cells (Figure 4K and Figure S4E). Altogether, these findings revealed a functional link between ALDH1A1 and different stages of metastatic dissemination.

### ALDH genes differently regulate PLK3 in RAR- and AR-dependent manner

Although *ALDH1A1* and *ALDH1A3* do not directly regulate gene expression, they synthesize retinoic acid (RA) from retinol and might regulate RA-dependent transcriptional programs through retinoid receptors. In the presence of RA, the retinoic acid receptor alpha (RARA) and the retinoid X receptor alpha (RXRA) transcription factors bind to RARE elements in the target gene promoters and regulate gene transcription. Two other retinoic acid receptors, RARG and RARB, exhibit a tissue-restricted pattern. In PCa, *RARB* expression is often lost due to promoter hypermethylation, whereas RARG was detected in PCa specimens 47 and is highly expressed in our PCa models.

**Figure 5:**
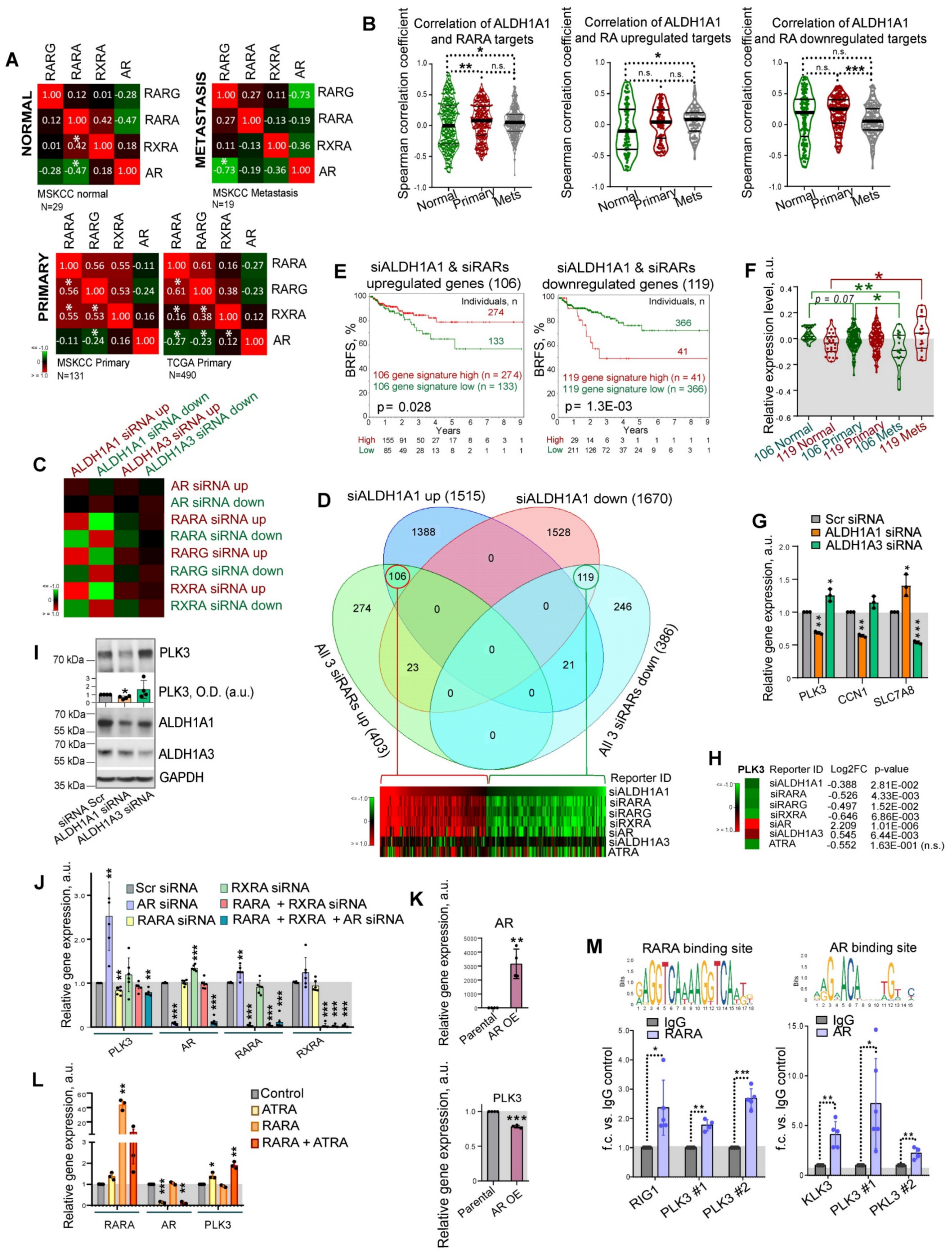
** ALDH genes differently regulate PLK3 in RAR- and AR-dependent manner.** (**A**) Correlation of RARG, RARA, RXRA and AR expression levels in noncancerous tissues (MSKCC dataset, n = 29), primary tumors (MSKCC dataset, n = 131; TCGA dataset, n = 490), and metastatic tumors (MSKCC dataset, n = 19); *p < 0.05. (**B**) Correlation of ALDH1A1 expression levels with the expression of the previously described RARA transcriptional targets 48 and genes reported to be up- or downregulated in response to RA treatment 48 in normal tissues (MSKCC dataset, n = 29), primary tumors (MSKCC dataset, n = 131), and metastatic tumors (MSKCC dataset, n = 19). Statistical analysis was performed by the Kruskal-Wallis rank sum test for multiple independent samples. Conover p-values were further adjusted by the Benjamini-Hochberg FDR method; *p < 0.05; **p < 0.01; ***p < 0.001. (**C**) An enrichment score calculation relative to randomly expected revealed similar gene deregulation after the knockdown of ALDH1A1 and each individual retinoid receptor. No such trend was found for genes deregulated after ALDH1A3 knockdown. (**D**) Venn diagrams showing specific and common significantly deregulated genes in response to the knockdown of ALDH1A1 and all 3 retinoid receptors (RARA, RXRA, RARG). (**E**) The Kaplan-Meier analyses of biochemical recurrence-free survival of patients with high (red) compared to the low (green) expression level of gene signatures, including either 106 genes upregulated or 119 genes downregulated after knockdown of ALDH1A1 and all 3 retinoid receptors (TCGA dataset). (**F**) A relative expression of 119 geneset and 106 geneset in noncancerous tissues, n = 29; primary tumors, n = 131; and metastases, n = 19 in the MSKCC dataset. Relative expression of genesets was calculated as median of quantile normalized gene expression levels; *p < 0.05; **p < 0.01. (**G**) RT-qPCR analysis of PLK3, CCN1, and SLC7A8 expression in LNCaP cells upon ALDH1A1 and ALDH1A3 knockdown. N = 3; Error bars = SD; *p < 0.05; **p < 0.01; ***p < 0.001. (**H**) The data of RNAseq analysis for the PLK3 regulation in response to the knockdown of ALDH1A1, ALDH1A3, retinoid receptors, or treatment with 5x10^-5^M of ATRA. (**I**) Western blot analysis of PLK3 protein levels after knockdown of ALDH1A1 or ALDH1A3 expression. Representative images of one of four independent repeats are shown. Error bars = SEM; *p < 0.05. (**J**) RT-qPCR analysis of PLK3, AR, RARA and RXRA expression in LNCaP cells upon either knockdown of AR, RARA, RXRA, or RARA and RXRA together or knockdown of all three genes. N ≥ 3; Error bars = SD; **p < 0.01; ***p < 0.001. (**K**) RT-qPCR analysis of PLK3 and AR expression in PC3 cells stably overexpressing AR. N ≥ 3; Error bars = SEM; **p < 0.01; ***p < 0.001. (**L**) RT-qPCR analysis of PLK3, AR, and RARA expression in LNCaP cells upon transient RARA overexpression, treatment with 50 µM of ATRA for 48 h, or both. Cells transfected with empty plasmid were used as control. N = 3; Error bars = SD; *p < 0.05; **p < 0.01; ***p < 0.001. (**M**) The results of chromatin immunoprecipitation (ChIP)-qPCR analysis in LNCaP cells confirmed the direct binding of RARA and AR proteins to the multiple promoter regions of the target gene PLK3. Corresponding IgG was used as a negative control. RARA and AR binding sites were taken from the JASPAR CORE database 94. N ≥ 3; Error bars = SD; *p < 0.05; **p < 0.01; ***p < 0.001.

Of note, retinoid receptors interplay with AR to regulate common target genes 14. In primary PCa gene expression datasets TCGA (N = 490) and MSKCC (N = 131), all retinoid receptors have a positive mutual correlation, and a weak negative or no correlation with *AR*. A negative association of *RARG* with *AR* increases in metastatic tumors (MSKCC cohort, N = 19), whereas the mutual correlation of *RARA* with *RARG* and *RXRA* decreases, suggesting a distinct role of these retinoid receptors in the metastatic transcriptional network (Figure 5A). An association of *ALDH1A1* with the RARA transcriptional program, including correlation with RARA targets and genes, known to be upregulated in response to RA 48, 49, increases in metastases compared to primary tumors and noncancerous tissues in the MSKCC dataset (n = 179). An opposite trend was observed for genes known to be downregulated after RA treatment (Figure 5B). In contrast, no cancer progression-related changes in correlation with RARA transcriptional program was found for *ALDH1A3* (Figure S5A).

To understand the mechanisms contributing to the differential roles of ALDH1A1 and ALDH1A3 in regulating PCa development, we used androgen-sensitive LNCaP cells for siRNA-mediated knockdown of *ALDH1A1*, *ALDH1A3*, *AR*, *RARA*, *RARG*, and *RXRA* genes or treatment with 5x10^-5^ M of ATRA for 48 h followed by RNA sequencing (RNAseq). A total of 3,185 genes were differentially expressed (p < 0.05) in cells after *ALDH1A1* knockdown, including 1,515 upregulated and 1,670 downregulated genes. An enrichment score calculation revealed that genes deregulated after ALDH1A1 knockdown are similarly regulated in response to the knockdown of each individual retinoid receptor, while no such trend was found for genes deregulated after *ALDH1A3* knockdown (Figure 5C). Among the 1,515 genes upregulated after *ALDH1A1* knockdown, 106 genes were also upregulated in response to the knockdown of all 3 retinoid receptors (*RARA, RXRA*,* RARG*), whereas 119 genes out of the 1,670 genes downregulated after *ALDH1A1* knockdown also downregulated after the knockdown of all 3 retinoid receptors (Figure 5D, Figure S5B). Of note, many genes commonly regulated after the knockdown of *ALDH1A1* and all 3 retinoid receptors are also significantly deregulated in the same direction after ATRA treatment (26 upregulated genes and 50 downregulated genes). Analysis of the TCGA PCa gene expression dataset confirmed that gene signatures including either 106 genes upregulated or 119 genes downregulated after knockdown of *ALDH1A1* and all 3 retinoid receptors have shown a significant correlation with either lower or higher BRFS, correspondingly (Figure 5E). A relative expression of 119 geneset increases in metastases compared to noncancerous tissues, whereas expression of 106 geneset is decreased in metastases compared to the primary tumor and noncancerous tissues in the MSKCC dataset (n = 179) (Figure 5F).

GSEA analysis suggested that gene signature similarly deregulated by ALDH1A1 and retinoid receptors is associated with BRCA1 signaling, cell response to the anti-proliferative and radiosensitizing drug CHR-2797 (tosedostat) 50, 51, and nucleolus functions (Figure S5C). Out of 33 genes similarly regulated by ALDH1A1, RARs and RXRA knockdown (22 genes upregulated and 11 genes downregulated), but oppositely regulated by ALDH1A3 (Figure S5D), several were chosen for independent verification by quantitative qRT-PCR and had similar gene expression patterns as in the RNAseq (Figure 5G). We next focused on one of the druggable targets, Polo-like kinase 3 (PLK3), a nucleolus protein involved in the cell cycle and DNA repair regulation. Depletion of *ALDH1A1* led to a decrease in the PLK3 gene and protein expression, while downregulation of the *ALDH1A3* gene increased the PLK3 gene and protein expression level (Figure 5H, I). First, we have confirmed PLK3 regulation by RARs and AR. Consistently with RNAseq results, the knockdown of *RARA* resulted in *PLK3* downregulation, whereas *AR* knockdown increased *PLK3* expression level (Figure 5J). A knockdown of *RXRA* resulted in *AR* upregulation, whereas *AR* knockdown significantly induced *RARA* expression suggesting a feedback mechanism. Negative regulation of *PLK3* by *AR* was additionally confirmed using PC3 cells stably overexpressing *AR* 52 (Figure 5K).

Positive regulation of *PLK3* by ATRA-dependent RARA transcription was confirmed by transient RARA overexpression in combination with ATRA treatment. A combination of RARA overexpression and treatment with 5x10^-5^ M of ATRA resulted in more potent stimulation of *PLK3* expression than ATRA treatment alone (Figure 5L). Additionally, we also confirmed the downregulation of *PLK3* in response to ALDH1A1 knockdown in 22Rv1 cells (Figure S5E). Interestingly, in contrast to LNCaP cells, *PLK3* expression is downregulated in 22Rv1 cells upon AR knockdown, confirming the interplay between the full-length AR, AR-V7, and potentially AR-regulated miRNAs in this cell line, as we discussed above.

Consistent with RNAseq data, the analysis of the PLK3 gene promoter revealed putative RARA 53 and AR 54 binding elements (Figure S5F). We next performed ChIP analysis with antibodies directed against total AR and RARA proteins. The previously described RARA and AR transcription targets, *RIG1* and *KLK3*, respectively, were used as a positive control 55, 56. Coverage of all predicted binding sites was achieved by employing multiple primer pairs for each gene promoter. Cell pre-treatment with 5x10^-5^ M of ATRA was used to induce RARA binding to RAREs in gene promoters 55. Our analysis revealed significantly increased precipitation of different promoter regions of *PLK3* with RARA and AR antibodies (Figure 5M) compared to the control IgG. These results suggest that *PLK3* is regulated by *ALDH1A1* and *ALDH1A3* genes in RAR and AR-dependent manner.

### PLK3 regulates PCa cell migration, proliferation and radioresistance

**Figure 6:**
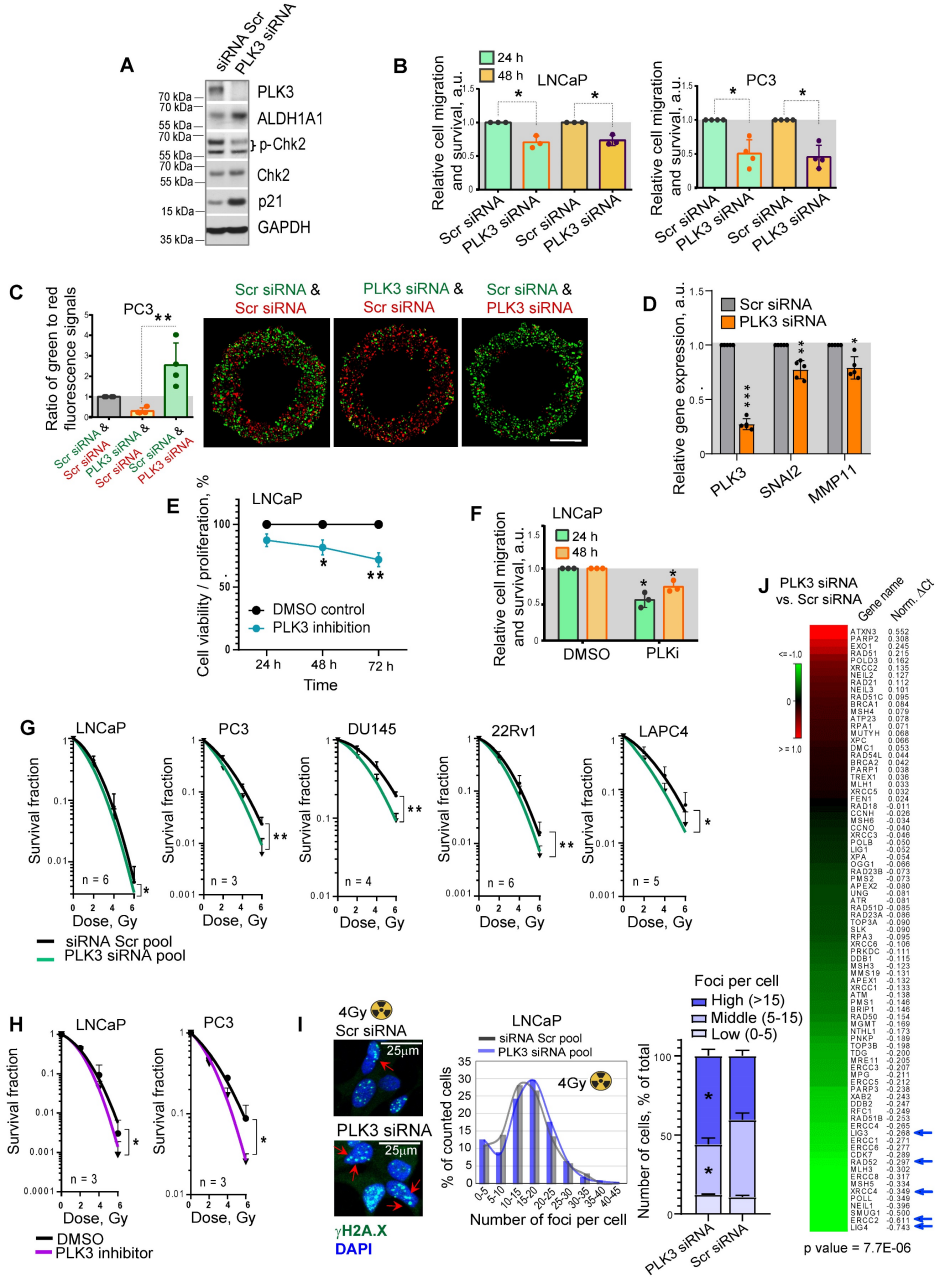
** PLK3 regulates PCa cell migration, proliferation and radioresistance.** (**A**) Western blot analysis of PLK3, ALDH1A1, Chk2, p-Chk2 (T68) and p21 expression after PLK3 knockdown; representative images of one of two independent repeats are shown. (**B**) Analysis of the relative cell migration and survival of LNCaP and PC3 cells upon PLK3 knockdown by using Oris migration assay. Cells transfected with Scr siRNA were used as a control. Cell invasion was analyzed 24 h and 48 h after cell plating. N ≥ 3; Error bars = SD; *p < 0.05. (**C**) Analysis of the relative cell migration and survival of PC3 cells stably expressing GFP or tdTomato after PLK3 knockdown by Oris migration assay. Cells transfected with Scr siRNA were used as a control. The intensity ratio of the green and red fluorescence was calculated within the invaded area 48 h after cell plating; Scale bars = 500 µm. N ≥ 3; Error bars = SD; **p < 0.01. (**D**) RT-qPCR analysis of SNAI2 and MMP11 expression in LNCaP cells in response to PLK3 knockdown. Cells transfected with Scr siRNA were used as a control; N ≥ 3; Error bars = SD; *p < 0.05; **p < 0.01; ***p < 0.001. (**E**) The CellTiter-Glo viability and proliferation analysis of LNCaP cells in response to the treatment with PLK3 inhibitor GW843682X at IC_50_ concentration of 1.73 x 10^-7^ M. N = 3; Error bars = SD; *p < 0.05; **p < 0.01. (**F**) Analysis of the relative cell migration and survival of LNCaP cells after 24 h pre-treatment with PLK3 inhibitor GW843682X at IC_50_ concentration of 1.73 x 10^-7^ M using Oris migration assay. Cells treated with DMSO were used as a control. Cell invasion was analyzed 24 h and 48 h after cell plating. N = 3; *p < 0.05. (**G**) Relative cell radiosensitivity was analyzed by 2D radiobiological colony forming assay after siRNA-mediated knockdown of PLK3 in LNCaP, PC3, DU145, 22Rv1 and LAPC4 cells. Cells transfected with scrambled (Scr) siRNA were used as control. N ≥ 3; Error bars = SD; *p < 0.05; **p < 0.01. (**H**) Cell radiosensitivity was analyzed after 24 h pre-treatment with PLK3 inhibitor GW843682X at IC_50_ concentrations in LNCaP cells (IC_50_ = 1.73 x 10^-7^ M), and PC3 cells (IC_50_ = 4.34 x 10^-7^ M). Cells treated with DMSO were used as control. N ≥ 3; Error bars = SD; *p < 0.05; **p < 0.01. (**I**) The knockdown of the PLK3 gene resulted in more severe DNA damage in LNCaP cells after irradiation. DNA double-stranded breaks (DSBs) were analyzed by γ-H2A.X foci analysis in the individual cells 24 h after 4 Gy of X-ray irradiation. Arrows show the exemplary γ-H2A.X foci; the graphs show a distribution of cell nuclei by foci number after 4Gy of X-ray irradiation. Scale bars = 25 µm; *p < 0.05. (**J**) PLK3 inhibition lowered the expression of crucial DNA damage response regulators. LNCaP cells transfected either with scrambled (Scr) siRNA or with PLK3 siRNA were analyzed by RT2 DNA Repair profiler qPCR assay.

In contrast to PLK1, which has been suggested as a potential target for therapeutic intervention of PCa and other types of malignancies 57, the data for the role of PLK3 in regulating PCa is still scarce. Previous studies demonstrated that PLK3 is required for G1/S cell cycle transition 58. Upon DNA damage, PLK3 mediates priming phosphorylation of Chk2 on S62 and S73 necessary for subsequent Chk2 phosphorylation on T68 by ATM and efficient activation of the DNA damage response 59. Indeed a knockdown of PLK3 resulted in the decrease of phospho-Chk2 (T68) level and accumulation of p21, similar to the effect from ALDH1A1 knockdown (Figure 6A, Figure 1H). A knockdown of PLK3 also induced upregulation of ALDH1A1, suggesting a balancing feedback loop. We next analyzed whether PLK3 expression affects the migration and proliferative potential of PCa cells by using Oris migration assay. We observed that genetic silencing of *PLK3* in LNCaP and PC3 cells resulted in decreased cell migration and proliferation 24 h and 48 h after cell plating (Figure 6B). To directly compare the migratory and proliferative capacity of cells with and without PLK3 knockdown, we performed the Oris migration assay with the color-coded PC3 cells used for the previously described zebrafish xenograft models. The equal numbers of GFP^+^ green and tdTomato^+^ red PC3 cells transfected with PLK3 or Scr siRNA were plated in the same well for Oris assay, and the intensity ratio of the green and red fluorescence was calculated within the area invaded after 48 h. Consistently with non-color-coded cells, we demonstrated that PLK3 downregulation decreased the migratory and proliferative potential of PC3 cells (Figure 6C). Knockdown of *PLK3* is associated with downregulation of the critical regulators of epithelial-mesenchymal transition and prognosticators of worse clinical outcomes in patients with PCa such as *SNAI2* and *MMP11* (Figure 6D). We next used a drugable approach to modulate PLK3 with a small-molecule inhibitor GW843682X. Chemical inhibition of PLK3 with IC_50_ concentration of GW843682X (1.73 x 10^-7^ M) led to decreased viability/proliferation and migration properties of LNCaP cells (Figure 6E, F, Figure S6A).

Radiobiological clonogenic analyses demonstrated that the knockdown of PLK3 in five cell lines results in PCa cell radiosensitization, disregarding the androgen sensitivity status (Figure 6G, Figure S6B,C). Similar results were obtained after PCa cell pretreatment with IC_50_ concentrations of GW843682X (1.73 x 10^-7^ M for LNCaP and 4.34 x 10^-7^ M for PC3 cells) (Figure 6H, Figure S6A,D). Consistently, the knockdown of the PLK3 gene resulted in more severe DNA damage in LNCaP and PC3 cells after irradiation with 4 Gy of X-rays (Figure 6I, Figure S6E). In line with this data, PLK3 inhibition lowered the expression of crucial DNA repair regulators, including *LIG4*, *ERCC2*, *XRCC4*, *RAD52*, and *LIG3* 60-63 (Figure 6J).

**Figure 7:**
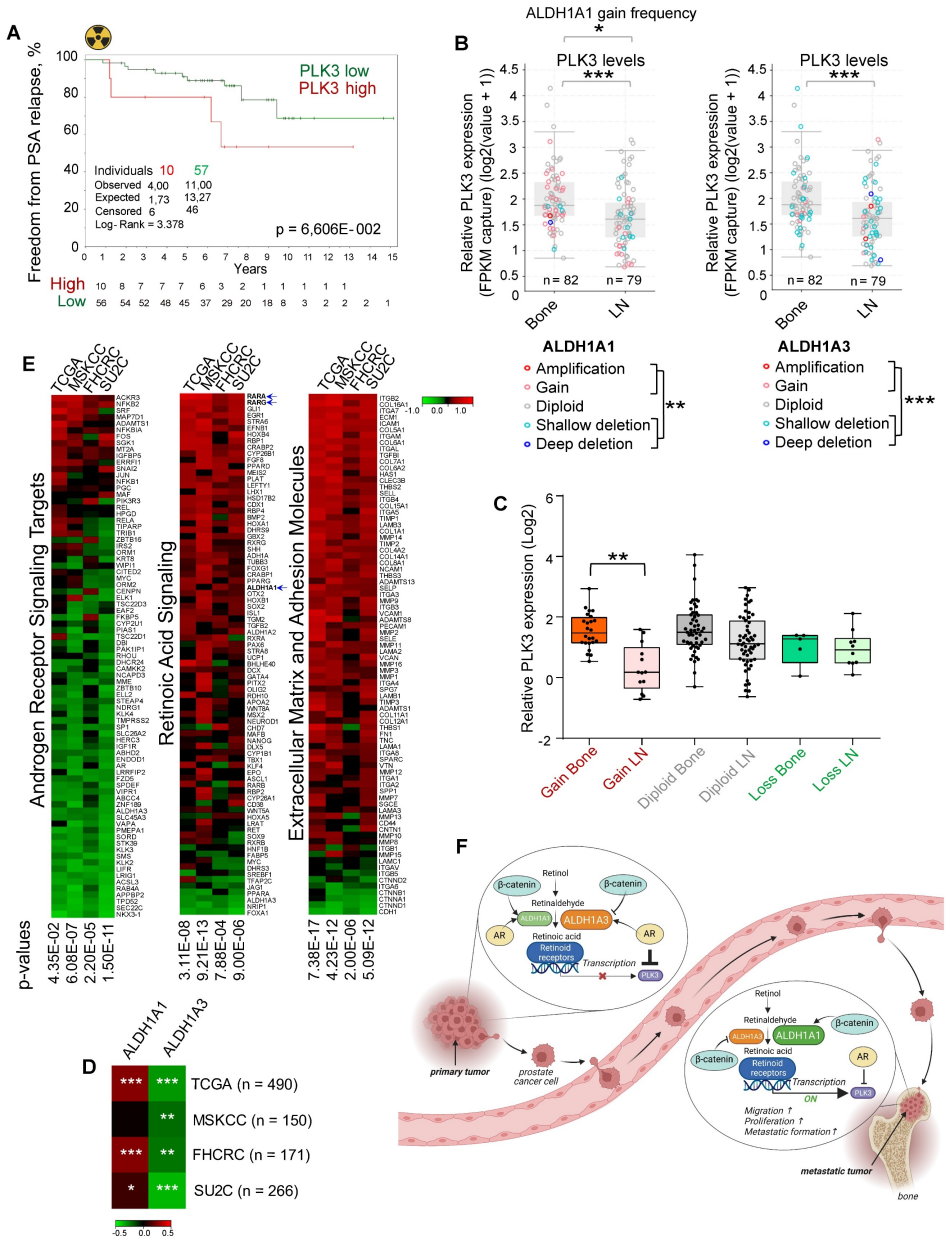
** PLK3 expression is associated with tumor resistance and metastases.** (**A**) The Kaplan-Meier analysis of freedom from PSA relapse in patients with prostate cancer treated with radiotherapy with high (red) compared to low (green) PLK3 expression levels. N = 67 (Dresden cohort). (**B**) ALDH1A1 gain and ALDH1A3 loss are associated with metastases. Relative PLK3 expression in higher in the bone metastases *p < 0.05; **p < 0.01; ***p < 0.001. Bone: bone metastases; LN - lymph node metastases. Analysis was performed using Metastatic PrCa (SU2C/PCF Dream Team, n = 161) 64. Statistical analysis was performed with Wilcoxon signed rank test with continuity correction. (**C**) Low PLK3 expression upon ALDH1A1 gain is found mostly in lymph node metastases but not in bone marrow metastases. Statistical analysis was performed by one-way ANOVA followed by posthoc Tukey's HSD (honestly significant difference) test; **p < 0,01. (**D**) Heatmap showing a correlation of ALDH1A1 and anti-correlation of ALDH1A3 with PLK3 expression in four prostate cancer datasets; *p < 0,05; **p < 0,01; ***p < 0.001. (**E**) Heatmap showing an anti-correlation of PLK3 expression with RT2 geneset for androgen receptor signaling targets, and positive correlation with expression of the extracellular matrix and adhesion molecules and retinoic acid signaling. Analysis was performed in four PCa datasets: TCGA (n = 490); MSKCC (n = 150); FHCRC (n = 171) and SU2C (n = 266). (**F**) PCa progression is associated with the increasing interplay of ALDH1A1 and RAR transcription program in regulating prostate cancer bone metastases and radioresistance in the AR-dependent manner. Created with BioRender.com.

To validate whether the *PLK3* expression levels correlate with tumor radioresistance in PCa patients, we analyzed the gene expression dataset for patients with PCa treated with radiotherapy (n = 67, Dresden cohort 9) and found a statistical trend for the association of high *PLK3* expression with lower relapse-free rates (p < 0.07) (Figure 7A). To understand the relevance of PLK3 for metastatic bone development, we analyzed the publicly available metastatic SU2C dataset 64. Consistent with our previous observations, we found that *ALDH1A1* gain and *ALDH1A3* loss are associated with metastases (Figure 7B). Furthermore, PLK3 has significantly higher expression in bone metastases than in lymph node metastases (Figure 7B), whereas low *PLK3* expression upon *ALDH1A1* gain is found mainly in lymph node metastases but not in bone marrow metastases (Figure 7C). Consistently, we found a correlation of *ALDH1A1* and anti-correlation of *ALDH1A3* with *PLK3* expression in four PCa datasets: TCGA (n = 490); MSKCC (n = 150); FHCRC (n = 171) and SU2C (n = 266) (Figure 7D). Interestingly, analysis of these four datasets revealed similar anti-correlation of PLK3 expression with androgen receptor signaling targets, and positive correlation with expression of the extracellular matrix and adhesion molecules, as well as genes involved in retinoic acid signaling (Figure 7E). Altogether, these findings suggest that ALDH1A1/PLK3 axis regulates the clinically relevant properties of PCa cells and is a potential druggable target for PCa management (Figure 7F).

## Discussion

Accumulating evidence in the field of cancer stem cell biology contributed to a changed view of high ALDH activity from an unobligated marker to a regulator of stemness in different types of tumors. A canonical function of ALDH metabolic enzymes is NAD(P)^+^ dependent oxidation of cellular aldehydes to carboxylic acids with generating NAD(P)H. The products of these reactions play an essential role in the maintenance of cellular homeostasis and survival. Some carboxylic acids produced by ALDH catalyzing reactions are bioactive metabolites such as a neurotransmitter γ-aminobutyric acid (GABA), and retinoic acid isomers such as ATRA and 9-cis RA serving as ligand for the RARs and RXRs transcription factors 65. The by-product of ALDH catalytic reaction, NADPH, plays a key role in the oxidative stress response by providing reducing equivalents for generating antioxidant molecules 66. Interestingly, NADPH has been suggested as a metabolic marker of CSCs 67.

To date, 19 ALDH isogenes have been identified in the human genome 12. In PCa, several ALDH isoforms, including ALDH1A1 and ALDH1A3 are highly expressed. However, not all of them equally contribute to the PCa progression and treatment outcome 12. High level of ALDH activity measured by conversion of bodipy-aminoacetaldehyde (BAAA) into bodipy-aminoacetate (BAA) is a marker of CSCs in different tumor types, including PCa. 9 out of the 19 ALDH proteins were suggested to contribute to this conversion 16. Our previous *in vivo* studies demonstrated that PCa cells with high ALDH activity are enriched for tumor-initiating populations and possess high radioresistance compared to their ALDH-negative counterparts 10, 11, 68. Here, we have shown an association of ALDH1A1 and ALDH1A3 isoforms with CSC phenotype and radioresistance in cell and animal models, and patients with PCa. However, we found a significant but opposite association of ALDH1A1 and ALDH1A3 expression with BRFS in patients with PCa treated with radiotherapy. These results suggest that ALDH1A1 and ALDH1A3 could play a differential role in the longitudinal tumor progression.

As essential signaling proteins for organogenesis, ALDH proteins are dynamically regulated during fetal and post-embryonic development 69. Furthermore, the expression level of ALDH1A1 protein in tumor tissues and its correlation with clinical outcomes in patients with breast cancer are age-dependent 70. We previously demonstrated that ALDH1A1 is dynamically regulated upon radiotherapy and upregulated in ALDH^+^ PCa cells reprogrammed from ALDH^-^ cells in response to *in vivo* tumor irradiation 10, 68*.*

In line with these findings, we found that ALDH1A1 and ALDH1A3 expression is dynamically changing during tumorigenesis. For the first time, we showed that the level of ALDH1A1 is upregulated in distant metastases compared to the primary tumor sites, and the expression of ALDH1A3 has an opposite regulation. Serial passaging of the metastasis-initiating cells in mice xenografts demonstrated a substantial increase in *ALDH1A1* expression in bone metastasis cells upon *in vivo* selection. These results suggest a role of *ALDH1A1* in the longitudinal evolution of bone metastasis-initiating properties. Furthermore, on the genomic level, both ALDH1A1 gain and ALDH1A3 loss are associated with bone metastases. Our study also showed that the expression level of ALDH1A1 in primary tumors negatively correlates with disease-free survival and clinical parameters associated with PCa aggressiveness, while a positive correlation was found for the ALDH1A3 gene.

This mutually exclusive expression of ALDH1A1 and ALDH1A3 can be attributed to their specific regulation either by β-catenin or by androgen 26. Indeed, we found a significant positive correlation of *ALDH1A1* with several gene sets related to cancer progression and metastatic dissemination, including WNT signaling, angiogenesis, osteogenesis, extracellular matrix, and adhesion. The experiments with the zebrafish xenograft model revealed decreased survival and extravasation of PCa cells upon β-catenin depletion, confirming a role of WNT/β-catenin in the metastatic dissemination. On the other hand, *ALDH1A3* was positively and *ALDH1A1* negatively associated with the expression of the AR transcription targets. Consistently, we observed a significantly increased iPSA level in patients with ALDH1A3 overexpressing tumors. In line with these observations, ADT was reported to reprogram bulk PCa cells into CSC populations 71. We also found that an association of these ALDH genes with the AR transcriptional program is dynamic in its nature: ALDH1A1 negatively correlates with AR target genes in noncancerous prostates; however this anticorrelation decreases in primary tumors and metastases. In contrast, ALDH1A3 has a high correlation with both AR expression and transcriptional program in normal tissues, however, it drops in primary tumors and even more decreases in metastases. PCa progression was associated with tumor dedifferentiation and gain of CSC features 6. In line with these findings, AR expression is low or lost in a substantial number of CRPC samples 72, and pathways other than AR signaling additionally contribute to disease progression in CRPC, such as WNT/β-catenin signaling 72, 73. Indeed, there is a reciprocal relationship between β-catenin and AR signaling, where AR negatively regulates the Wnt pathway, and WNT/β-catenin signaling can compensate for the loss of AR transcription 72, 74, 75. The WNT/β-catenin signaling pathway is highly activated in castration-resistant tumors, including AR-negative PCa 24, 72, 76, 77, whereas β-catenin inversely correlates with AR nuclear accumulation in PCa bone metastases 77.

Based on these observations, we hypothesized that AR- and β-catenin-regulated ALDH1A1 and ALDH1A3 expression is not only the marker but a mediator of PCa metastatic development. PCa cells with high ALDH activity were previously characterized as a population with high metastasis-initiating properties 78. However, until now, the contribution of individual ALDH isoforms to PCa metastasis development remains unclear. We hypothesized that ALDH proteins could regulate tumor dissemination and therapy resistance through activation of the specific transcriptional program by retinoic acids as products of ALDH enzymatic activity.

The transcriptional program activated by retinoic acids plays a special role in prostate development and functional maintenance as it antagonizes AR in the regulation of some prostate-specific genes such as human transglutaminase (hTGP) and prostate-specific antigen (PSA) 14, 49. Retinoic acid isomers such as ATRA affect transcriptional regulation by binding to the nuclear retinoic acid receptors (RARs) and the retinoid X receptors (RXRs). RARs function as transcriptional regulators in the form of heterodimers with RXRs, whereas RXRs can activate transcription as homodimers. The dimers of RXRs and RARs bind to the specific RARE DNA sequences and regulate target gene transcription once loaded with retinoid ligands. Different retinoic acid isomers bind and transcriptionally activate RAR/RXR complexes. Although the efficacy and specificity of transcriptional stimulation is isomer-dependent, ATRA serves as pan-agonists of RARs 79, 80. The biological role of the individual retinoid receptors depends on the tissue-specific crosstalk with other transcriptional regulators such as AR in prostate tissues 49 and estrogen receptor in breast epithelial cells 48. All retinoid receptors described in this study, RARA, RARG, RXRA, are expressed in normal and cancerous human prostate tissues 47 and are reported to interplay with AR transcriptional activity 14, 49. This study revealed that *AR* and *RARA* gene expression is mutually exclusive, and activation of RARA-dependent transcription inhibited *AR* mRNA level in PCa cells. We also found that retinoid receptors and AR cooperatively regulate common target genes: 202 genes similarly regulated after the knockdown of ALDH1A1 and all 3 retinoid receptors are also deregulated in the same direction after AR knockdown, while only 23 had an opposite direction.

Among the genes that showed similar regulation by knockdown of ALDH1A1 and retinoid receptors, and the opposite regulation by ALDH1A3, we selected PLK3 as one of the druggable targets reported to be associated with PCa progression. Furthermore, analysis of the metastatic PCa dataset showed that the gain of ALDH1A1 copy number in PCa bone metastases is associated with high PLK3 expression. We have validated that AR and RARA oppositely regulated PLK3, and this regulation is mediated by the direct binding of these transcription factors to the PLK3 promoter. PLK3 regulates the cell entry into the S phase and DNA double-strand break repair by phosphorylation of the BRCA1 interacting protein, CtIP 81. Of note, by GSEA analysis, we found the BRCA1 network in close association with the set of genes deregulated by the knockdown of ALDH1A1 and retinoid receptors. The role of PLK3 in carcinogenesis depends on tumor type 82. It has been described as a positive regulator of proliferation and migration in PCa 83. PLK3 contributes to the regulation of critical cellular processes, including DNA damage response and cell cycle control 84. Our study employing PLK3 knockdown or chemical inhibition confirmed its role as a regulator of PCa cell proliferation and migration. Knockdown of PLK3 inhibited expression of MMP11 and SNAI2, known players in the regulation of cancer invasion and metastasis 85. Interestingly, our recent study revealed that SNAI2 contributes to the radiation-induced PCa reprogramming, and SNAI2 knockdown reduced the expression of ALDH1A1 and increased ALDH1A3 expression in PCa cells 68. SNAI2 was recently described as a negative regulator of PCa sensitivity to ADT 86. Furthermore, we found that PLK3 knockdown lowered the expression of crucial DNA damage response genes and is associated with a higher number of the residual γH2A.X foci after irradiation, a marker of the unrepaired DNA double-strand breaks. Both the knockdown of PLK3 and its chemical inhibition resulted in PCa cell radiosensitization. In line with these *in vitro* observations, we also found a close to significant association of high PLK3 expression with worse BRFS in patients with PCa treated with radiotherapy in our institute. Thus, we demonstrated that the PLK3 gene contributes to the control of PCa cell proliferation, migration, DNA repair, and cell radioresistance, and its expression is positively regulated in the ALDH1A1/RARA-dependent manner and negatively regulated by AR.

Much preclinical evidence confirms the link between DNA repair and tumor metastases. Metastatic tumor dissemination is a complex process, and each stage of a tumor cell's journey to the metastatic site might be associated with DNA damage. Constricted tumor cell migration, which mimics tumor cell spreading from a localized tumor and extravasation to the metastatic site, is associated with nuclear envelope rupture (NER), and, consequently, increased DNA damage and repressed cell cycle and proliferation 87. Furthermore, due to their detachment from ECM, shear stress, and increased oxygen concentration in the bloodstream, circulating tumor cells (CTCs) produce high levels of reactive oxygen species (ROS) associated with higher levels of DNA damage and, consequently, activation of the DDR signaling 88. Notably, previous research revealed increased levels of ALDH positive cells after cancer cell exposure to the flow-based shear stress similar to it in the circulation. This data suggested the role of ALDH proteins in the shear stress-protecting mechanisms 89. Thus, efficient ROS scavenging and DNA damage repair are prerequisites for tumor cell survival during metastatic spread and their re-entering of a proliferative pool at the metastatic site.

To our knowledge, this is the first report revealing the role of the CSC regulators ALDH1A1 and ALDH1A3 in the AR-dependent gene expression. This function is mediated by their interplay with AR through the RAR-dependent transcriptional program. In addition, we found that ALDH1A1 and ALDH1A3 play opposite roles in the regulation of PCa metastases in the experimental *in vivo* models, with ALDH1A1 being a positive regulator and ALDH1A3 being an inhibitor of metastatic dissemination. We have confirmed our findings by a comparative analysis of the expression levels of ALDH1A1 and ALDH1A3 proteins in benign, primary, metastatic PCa tissues and locally recurred tumors. We also validated ALDH1A1 and ALDH1A3 as potential biomarkers of clinical outcomes and metastases in the cohort of patients with PCa. Despite the previous clinical studies from our team confirming the effect of ablative radiotherapy (aRT) on local control of prostate bone metastases in oligometastatic PCa 90, a subset of patients does not respond, and the disease progresses further. To date, there is no biomarker available to identify these patients. This report provides the first evidence that ALDH1A1 and PLK3 could potentially serve as prognostic biomarkers for patients with PCa treated with radiotherapy and potential targets to eliminate metastasis-initiating and radioresistant tumor cell populations. There are currently no specific ALDH1A1 and PLK3 inhibitors available for clinical cancer treatment. However, some non-cancer drugs could be repurposed for this treatment. For example, Disulfiram used for the treatment of chronic alcoholism, has been currently tested for targeting ALDH activity in glioblastoma 91 (NCT01777919, NCT02715609). Our previous findings revealed that inhibition of the β-catenin signaling pathway and epigenetic therapies are promising strategies to eradicate ALDH positive populations in PCa models 10, 11, 40. Our current findings suggest that bisphosphonates such as zoledronic acid inhibit *ALDH1A1* expression in a dose-dependent manner. Future research is needed to test these inhibitors in combination with radiation therapy in the preclinical metastatic PCa models and to validate prognostic values of the identified biomarkers in the prospective clinical study. Importantly, due to differences and impact of androgen levels between the species, an extrapolation of findings from zebrafish and mouse studies directly to humans should be used cautiously. Employing patient-derived *ex vivo* models such as organs-on-a-chip providing physiologically relevant microenvironment 92 could be an alternative approach to further validate these findings.

## Materials and Methods

Additional methods not described here are included in the Supplementary information. Clinicopathological characteristics of PCa patients are described in Supplementary Table S1. shRNA constructs used for the knockdown of Aldh1a1 and Aldh1a3 are described in Supplementary Table S2. Antibodies, primers, and siRNA oligonucleotides used for the study are described in Supplementary Table S3. Geneset lists are included in Supplementary Table S4.

### Clinical specimens

Clinical material was collected with informed consent from all subjects. The ethical approvals for these retrospective analyses of clinical and biological data were obtained from the respective local Ethics Committees. Benign samples, primary PCa samples, and locally recurrent or locally advanced tumors used for the immunohistochemical staining in this study were from patients diagnosed with PCa in the Hospital of Göppingen, Germany between 1997 and 2014. Lymph node and distant metastases are from patients treated in the University Hospital Schleswig-Holstein, Campus Lübeck between 2002 and 2015 (Lübeck cohort). For the evaluation of ALDH1A1 and ALDH1A3 expression in human PCa specimens, 613 and 325 samples were stained for ALDH1A1 and ALDH1A3, respectively. Among them, 33 (ALDH1A1) / 17 (ALDH1A3) benign prostatic samples, 457 (ALDH1A1) / 170 (ALDH1A3) primary PCa samples obtained by radical prostatectomy, 55 (ALDH1A1) / 52 (ALDH1A3) local recurrent or locally advanced PCa samples obtained by transurethral resection of the prostate, 35 (ALDH1A1) / 29 (ALDH1A3) lymph node metastases and 33 (ALDH1A1) / 57 (ALDH1A3) distant metastases were analyzed. 457 primary tumor samples are from 215 patients since we included up to 7 different tumor foci per patient. For 205 patients who underwent curative intended surgical tumor resection, the follow-up data was available to perform Kaplan-Meier analysis. Disease recurrence was defined as rising serum PSA level after radical prostatectomy indicating disease progression.

In the current study, we also concerned the patient cohort that included 95 prostate cancer patients referred to robot-assisted laparoscopic radical prostatectomy (RALP) at Oslo University Hospital between October 2011 and May 2016 (Oslo cohort). All patients were enrolled in the FuncProst study (NCT01464216), and detailed clinical characteristics were described previously 29. The following clinical parameters were taken for the correlation analysis: age at inclusion, cT1vsCT2vcCT3 (grouped clinical tumor stages), Glscorepat (Gleason score determined by pathologists after surgery), largest extent (largest extent histologically determined by the pathologists based on HE-stained whole-mount sections), N status pato PSA (lymph node status of patients; determined by pathological examination of lymph nodes when pelvic lymph node dissection (PLND) was performed. In patients without PLND, negative MRI in combination with undetectable PSA at 6 weeks after surgery was regarded as nodal stage 0), PSA (PSA measured before surgery); pT2vs3vs4 (grouped pathological tumor stage); risk classification (D'amico risk classification: 1 = low, 2 = intermediate, 3 = high) 29.

In addition, a whole transcriptome analysis was performed using the HTA 2.0 Array (Affymetrix) using formalin-fixed paraffin-embedded (FFPE) tumor tissues of patients with intermediate- or high-risk localized PCa (n = 67) treated with curatively-intended, definitive radiotherapy at the Department of Radiotherapy and Radiation Oncology, University Hospital Carl Gustav Carus and Faculty of Medicine, Dresden (Dresden cohort). Patient clinical characteristics are described previously 9. The clinical endpoint was freedom from PSA relapse. Survival curves were estimated by the Kaplan-Meier method.

### Immunohistochemistry

ALDH1A1 and ALDH1A3 protein expression was detected and quantified using immunohistochemistry (IHC). Fresh frozen FFPE tissue blocks (donor blocks) were used to create tissue microarrays (TMA). Three representative cores per sample from donor blocks were placed into a TMA recipient using a semiautomated tissue arrayer (Beecher Instruments, Sun Prairie, WI, USA). Immunohistochemistry was performed after deparaffinization, following treatment with a primary anti-ALDH1A1-antibody (Thermo Fisher Scientific, PA5-11537) or anti-ALDH1A3-antibody (Atlas Antibodies, HPA046271) on the Ventana BenchMark (Roche, Basel, Switzerland) by using the IView DAB Detection Kit. Expression levels were evaluated by two pathologists (AO, SP) and categorized according to negative, low to moderate, and high staining intensity. The protein expression in all three replicates per sample was considered, and the highest expression in a single core was used for further analysis in cases of heterogeneous staining levels. Androgen receptor (AR) expression was detected and evaluated as described before 93.

### Statistical analysis

The results of the flow cytometry analyses, densitometry data generated for western blots, sphere formation assay, ChIP analysis, cell migration, viability assays, and relative gene expression determined by qPCR were analyzed by paired two-tailed t-test. Statistical analysis for the zebrafish xenograft experiments and analysis of γH2A.X in the individual tumor cells was performed using an unpaired two-tailed t-test. Additional information about specific statistical analyses is included in the figure legends. Sample sizes were determined based on previous studies involving similar experimental setups, and at least three biological repeats of each experiment were performed. The cell survival curves were analyzed using SPSS v.23 software by linear-quadratic formula S(D)/S(0) = exp(-αD-βD2) using stratified linear regression after transformation by the natural logarithm. A significant difference between two survival curves was determined by GraphPad Prism software v.8. A significant difference between the two conditions was defined as *p < 0.05; **p < 0.01; ***p < 0.001. The correlation of gene expression levels was evaluated by SUMO software using the Pearson or Spearman (for nonparametric data) correlation coefficient. For *in vivo* mouse experiment, outliers were removed by the iterative Grubbs' method with α = 0.05. IC_50_ values (50% inhibitory concentrations) were determined by non-linear regression using GraphPad Prism software.

## Supplementary Material

Supplementary methods, figures and tables.

## Abbreviations

ALDH: aldehyde dehydrogenase
AR: androgen receptor
ATRA: all-trans retinoic acid
BRFS: biochemical recurrence free survival
CSC: cancer stem cells
CRPC: castration-resistant prostate cancer
MIC: metastasis initiating cells
PLK3: polo-like kinase3
PSA: prostate specific antigen
RARA: retinoic acid receptor alpha
RARG: retinoic acid receptor gamma
RXRA: retinoid X receptor alpha
