# Supplementary material for: ALDH1A1 drives prostate cancer metastases and radioresistance by interplay with AR- and RAR-dependent transcription
Source: Theranostics. 2024 Jan 1;14(2):714–37. doi: 10.7150/thno.88057 (PMC10758061; doi:10.7150/thno.88057)
Supplement: Supplementary file 1 — Supplementary methods, figures and tables. [file thnov14p0714s1.pdf]

Supplementary Information

Supplementary Methods

Cell lines and culture condition

PCa cell lines PC3 (derived from bone metastasis), LNCaP (derived from lymph node metastases), LNCaP-C4-2B (further named C4-2B, a bone metastatic derivative subline of human prostate cancer LNCaP cell line), DU145 (derived from a brain metastasis lesion), LAPC4 (derived from lymph node metastasis), 22Rv1 (derived from an androgen-dependent CWR22 xenograft tumor, which was regressed and relapsed after castration, and serially passaged in mice) were purchased from the American Type Culture Collection (Manassas, VA, USA) and cultured according to the manufacturer's recommendations in a 37°C incubator in an atmosphere with 5% CO_2. PC3 cells overexpressing AR was a kind gift of Dr. Andy Cato (Karlsruhe Institute of Technology (KIT), Germany) and established as described previously [1]. PC3 and DU145 cell lines were cultivated in DMEM medium (Sigma-Aldrich); LNCaP 22Rv1, LAPC4 and C42B cells were grown in RPMI1640 medium (Sigma-Aldrich). Cell medium was supplemented with 10% FBS (Sigma-Aldrich) and 2 mM L-glutamine (Sigma-Aldrich). LAPC4 cells were cultured in a medium supplemented with 100 nM dihydrotestosterone (DHT); the medium was changed every 3 days. Radioresistant (RR) cell lines were established as described before [2, 3]. Cell radioresistance was verified by radiobiological clonogenic cell survival assay before experimentation. Corresponding age-matched non-irradiated parental cells were used as controls for RR cell lines. The murine prostate carcinoma cell line RM1 bone metastatic (BM) expressing GFP was a kind gift of Dr. Power (University of New South Wales, Australia) and established as was described previously [4]. RM1(BM) cells were cultured in RPMI1640 medium (Sigma-Aldrich) supplemented with 10% FBS (Sigma-Aldrich) and 2 mM L-glutamine (Sigma-Aldrich).

Establishment of color-coded PC3 cell lines

Color-coding of PC3 cell line with green or red fluorescent proteins was performed using the pWPXL vector and its derivative construct, where EGFP was replaced by tdTomato. HEK293 cells were transfected by these constructs along with psPAX2 and pMD2.G plasmids using the calcium phosphate method to produce replication-incompetent lentiviral particles. Supernatant from transfected cells was collected for 3 days, pooled, cleared through 0.45 µm filter and applied on PC3 cells overnight. Transduced PC3 cells were passaged twice to expand and eliminate any residual lentivirus, and after that, populations stably expressing corresponding fluorescent proteins were isolated via fluorescence-activated cell sorting (FACS).

shRNA-mediated gene silencing

RM1(BM) cells were transfected with pLKO.1 puro vector constructs expressing shRNA against mouse *Aldh1a1, Alhd1a3* or nonspecific control shRNA (shNS) using Lipofectamine 2000 Transfection Reagent (Thermo Scientific, Waltham, MA) according to the manufacturer’s instructions. 48 h after transfection, cells were selected with puromycin at 4 µg/ml concentration until discrete colonies appeared. The list of shRNA constructs is provided in Supplementary Table 2.

siRNA-mediated gene silencing

The cells were grown until 60-80% confluency in a complete medium. The Lipofectamine RNAiMAX (Thermo Scientific, Waltham, MA) and siRNAs were diluted at the corresponding concentrations in Opti-MEM reduced serum medium and used for cell transfection according to the manufacturer's instructions. After adding the transfection reagents, the cells were incubated at 37°C in a CO_2 incubator for 48 h. Cells transfected with unspecific siRNA (scrambled siRNA or siSCR) were used as a negative control in all knockdown experiments. The siRNA sequences used in the study are provided in Supplementary Table 3.

Transient gene overexpression

LNCaP cells were transfected with plasmid DNA, including pEGFP (Clontech) and pcDNA3.1-hRARα (Addgene # 135397, a gift from Catharine Ross [5]) using Xfect™ DNA Transfection Reagent (Takara Bio) according to the manufacturer's instruction. The cells were treated with $5x10^{-5}$M of ATRA for 48 h, followed by RT-qPCR analysis. pEGFP expression was used for the analysis of transfection efficiency.

PCa cell preparation for injection into 2 days post fertilization (dpf) zebrafish larvae

PC3 color-coded cells (GFP or tdTomato) were trypsinized (0.25 % trypsin-ethylenediaminetetraacetic acid, Gibco) with the subsequent addition of growth media to stop the reaction. The cell suspensions were centrifuged at 1200 rpm for 5 min, and the pellets were resuspended in PBS to achieve $1x10^6$ cells/ml. Next, the suspensions were transferred into a 1.5 ml Eppendorf tube and centrifuged at 800 rpm for 10 min. Afterward, cancer cells were washed once in PBS and one time in Tx Buffer (PBS, 1% Penicillin/Streptomycin, 1.5 µM EDTA). Finally, the pellet of $1x10^6$ cells was resuspended in 10 µl Tx Buffer.

Injection of PCa cells into 2 dpf zebrafish embryos

Adult zebrafish of the strain flk1:CFP (Tg(*kdrl*:CFP)^{zf410})[6] were incrossed for the generation of embryos with a vessel marker. Subsequently, eggs were collected, selected for CFP+ signal using stereomicroscope (Leica MZ16 FA), transferred to a 10 cm plastic dish and kept in E3-medium (5 mM NaCl, 0.17 mM KCl, 0.33 mM CaCl2, 0.33 mM MgSO4) at 28°C in an incubator (TS608/2-1, WTW). 2 dpf embryos were mechanically dechorionated with sharp tweezers and anesthetized with 0.02% Tricaine solution (MS222, Merck) in a plastic dish. The larvae were transferred on a grooved 1.5% agarose bed cast before experiments. Fine borosilicate glass tubes with filaments were pulled into injection capillaries by Flaming/Brown Micropipette Puller (Model P-97, Sutter Instruments Co.). The capillary diameter was adjusted to 20 µm by cropping it with a tweezer. Cells were mixed immediately before injection again by repeated pipetting. 6 µl of cell suspension was loaded into the capillary, which in turn was inserted into a microinjector (MM3301R, Marzhauser). The final injection volume was adjusted to 4 nl (to introduce around 400 cells in total), and cells were injected into the Duct of Cuvier (DoC) of anesthetized zebrafish embryos with the help of a pneumatic pump (Pneumatic PicoPump PV 820, WPI) and binocular (SZX10, Olympus). The fish were transferred to a new 10 cm dish containing fresh E3-medium at a density of up to 50 embryos per dish and incubated at 33°C until 5 dpf.

Imaging of injected zebrafish larvae

At 5 dpf, 1 ml of 1% low melting point agarose (Biozym Scientific) aliquots were melted in a water bath at 68°C. Upon melting, tubes were transferred to a 42°C water bath and 20 µl of 0.4% Tricaine solution were added per aliquot. Meanwhile, zebrafish larvae were anesthetized with 0.02% Tricaine solution added to a 10 cm dish containing E3-medium.

Immersed in agarose, injected larvae were transferred on microwell dishes with a transparent glass bottom (35 mm with 14 mm microwell, MatTek Corporation). After solidifying of the agarose, E3-medium with 0.01% Tricaine was carefully added until the agarose was fully submerged. Subsequently, the tail regions of the fish were imaged with a Dragonfly Spinning Disc Confocal Microscope (Andor Technology). Extravasation and survival were assessed using Fiji software.

Clonogenic cell survival assay

Radiobiological clonogenic assay was performed as described previously [7]. Cells were plated at a density of 1000-4000 cells/well depending on the cell line and treatment in 6-well plates in triplicates. The following day cells were irradiated with doses of 2, 4 and 6 Gy of X-rays (Yxlon Y.TU 320; 200 kV X-rays, dose rate 1.3 Gy/min at 20 mA) filtered with 0.5 mm Cu. The absorbed dose was measured using a Duplex dosimeter (PTW). Cells were incubated in a humidified 37°C incubator supplemented with 5% CO_2 to form colonies. 10 days later, the colonies were fixed with 10% formaldehyde (VWR International) and stained with 0.05% crystal violet (Sigma-Aldrich). Colonies containing >50 cells were counted using a stereomicroscope (Zeiss). The plating efficacy (PE) at 0 Gy and surviving fraction (SF) were calculated as described previously [7].

Sphere forming assay

Cells with or without treatment were plated as single cell suspension at a density of 2000-5000 cells/well depending on the cell line in 24-well ultra-low attachment plates in Mammary Epithelial Cell Growth Medium (MEBM) (Lonza, Germany) supplemented with 4 µg/ml insulin (Sigma-Aldrich), B27 in a dilution 1:50 (Invitrogen), 20 ng/ml epidermal growth factor (EGF) (Peprotech), 20 ng/ml basic fibroblast growth factor (FGF) (Peprotech). Spheres were analyzed 14 days after cell plating. Cell clusters were disaggregated by pipetting before analysis. Plates were automatically scanned using the Celigo S Imaging Cell Cytometer (Nexcelom). The number and size of spheres were analyzed using ImageJ 1.8.0 software. A complementary cumulative distribution function was used for the analysis of the number and size of tumor spheres. Cell aggregates were discriminated from spheres based on their shape, size, and structure and excluded from the analysis.

RNA isolation, cDNA synthesis, and RT-qPCR

RNA from PCa cells was isolated by RNeasy Mini kit Plus (Qiagen) according to the manufacturer's recommendations. Reverse transcription was done using the PrimeScript™ RT reagent Kit (Takara) according to the manufacturer's recommendation. The volume of RNA for reverse transcription was adjusted in all samples to obtain a unified RNA concentration. Minus reverse transcriptase sample (-RT) was used as technical control. Quantitative real-time polymerase chain reaction (qRT-PCR) was carried out using the TB Green Premix Ex Taq II (Takara Bio Inc) according to the manufacturer's protocol for a total reaction volume of 20 µl. The qPCR cycling program was set on a StepOnePlus system (Applied Biosystems): 94°C for 30 min, 40 cycles: 94°C for 15 sec, 58°C for 60 sec, 72°C for 60 sec followed by a melt curve to 95°C in steps of 0.3°C. All experiments were conducted using at least three technical replicates. The expression of ACTB and RPLP0 mRNA was used as a reference to internal control for data normalization depending on the experiment. The primers used in the study were synthesized by Eurofins Genomics Germany GmbH and are listed in Supplementary Table 3.

Chromatin immunoprecipitation (ChIP)

ChIP experiments were performed with the Chromatin Immunoprecipitation (ChIP) Assay Kit (Merck Millipore) according to the manufacturer`s instructions. First, the DNA fragmentation step was conducted using Micrococcal Nuclease (Cell Signaling Technology) according to the ChIP protocol of the manufacturer. In brief, $2x10^6$ LNCaP cells were treated with 50 µM of ATRA. 48 h after the treatment start, proteins were cross-linked to the DNA by incubation with 1% formaldehyde (Thermo Fisher Scientific) for 10 min at 37°C. Cells were collected in PBS containing protease inhibitor cocktail (Cell Signaling Technology) and lysed in the recommended buffers A and B from the Cell Signaling Technology protocol. Next, nuclear DNA was fragmented by incubating nuclei with 0.125 µl Micrococcal Nuclease (Cell Signaling Technology) for 10 min at 37°C. After that, nuclei were transferred to SDS Lysis buffer (Merck Millipore), and the following steps were performed according to the Merck Millipore ChIP protocol. Disruption of nuclei was achieved by repeatedly passing the suspension through a syringe with a needle of 27G size (0.4 x 19 mm). Then, samples were incubated with primary antibody against AR (Cell Signaling Technology) and RARA (Cell signaling technology) or control Rabbit IgG antibody (Cell Signaling Technology) overnight at 4°C. On the next day, DNA-protein-antibody complexes were precipitated using Agarose beads, and crosslinks were reversed at 65°C for 4 h. The DNA fragments were further purified using the QIAquick PCR Purification Kit (Qiagen). For qPCR detection of immunoprecipitated DNA fragments, primers were designed to cover different promoter regions that contained putative AR or RARA binding sites (predicted by Eukaryotic Promotor Database, https://epd.epfl.ch//index.php).

Oris migration assay

Oris migration assay was used to validate the ability of the cells to migrate *in vitro.* Firstly, 200.000 cells/well were seeded in 6-well plates and were incubated for 24 h at 37˚C, 5% CO_2. 24 h later, cells were trypsinized and plated in 96-well collagen I-coated plates at a density of 30.000 cells/well. The silicon stoppers were inserted into the wells to keep the center of the well free of cells before the start of migration analysis. After 24 h, stoppers were removed, and cells were scanned using the Celigo S Imaging Cell Cytometer (Nexcelom) pre-migration (t = 0 h). The plate was then incubated for 24 h – 48 h to permit the migration into the central zone of the wells, and then was scanned post-migration. The pictures were analyzed by ImageJ software, and the area invaded by cells within this time was compared.

Luciferase reporter assay

The cells were seeded in a 96-well black/clear bottom plate with a density 30 000 cells/well. Next, the cells were serum starved in RPMI with 3% FBS for 24 h. Cells were subsequently treated with Zoledronic acid (Zol) (Cayman Chemical) at concentrations 100 µM, 50 µM or PBS as the control and co-transfected with pEGFP-N3 plasmid and ALDH1A1-promoter plasmid DNA or empty plasmid using Xfect™ Transfection Reagent (Takara Bio) according to the manufacturer's instruction. ALDH1A1-promoter luciferase reporter and empty plasmid were purchased from Switchgear Genomics. DNA for pEGFP-N3 was obtained from Clontech. Luciferase assay was conducted using LightSwitch Assay reagents (Switchgear Genomics). Luciferase activity was normalized to the GFP fluorescent intensity.

Aldefluor™ assay and flow cytometry

Aldehyde dehydrogenase activity was analyzed using the Aldefluor™ assay, according to the manufacturer's protocol (Stem Cell Technologies). In brief, cells were detached using Accutase (Sigma-Aldrich), washed with PBS, and resuspended in Aldefluor buffer. Cells

were incubated with the specific ALDH inhibitor diethylaminobenzaldehyde (DEAB) in concentration 1:50, which served as a negative control. Both control and positive samples were then stained with Aldefluor reagent at the concentration 1:200 and incubated at 37°C for 30 min. Dead cells were excluded after the staining with 1 µg/ml propidium iodide (PI), and doublets were excluded using the FSC-W and SSC-W functions of the BD FACSDiva 8.0.1 software. Stained cells were excited with a blue laser (488 nm), and the analysis was performed using the FITC channel. Samples were analyzed with the BD Celesta flow cytometer. A minimum of 100.000 viable cell events was collected per sample. Data were analyzed using FlowJo 10.7 software, and gates were set according to the DEAB control.

Chemical treatment

Enzalutamide, XAV939, Zoledronic acid (Zol) were purchased from Cayman Chemical Company, All-trans-retinoic acid (ATRA) from Sigma-Aldrich and GW843682X from Biomol GmbH. DMSO was used as a drug solvent for Enzalutamide, XAV939, ATRA, GW843682X, and corresponding concentrations of DMSO were used as controls in all the experiments, which included cell treatment. PBS was used as a drug solvent for Zol and as a control. The cells were serum starved in DMEM or RPMI with 3% FBS for 24 h followed by treatment with XAV939 antagonist at concentration 10, 50 and 100 µM and with Enzalutamide inhibitor at concentrations 5, 10 and 20 µM for 48 h. For Zol treatment experiments, the drug was used in concentrations 50 and 100 µM for 48 h. For ATRA treatment, the drug was used in concentrations 10, 25 and 50 µM for 48 h. For GW843682X treatment experiments, the inhibitory concentration (IC_{50}) was determined (LNCaP, IC_{50}=1.731E-007 M; PC3, IC_{50}=4.337E-007 M). After 48 h in the incubator, cells were used for functional assays or RNA isolation. In total, at least three independent biological repeats were performed with cells at different passages.

Inhibitory concentration (IC_{50}) determination

To determine the IC_{50}, 200.000 cells/well were seeded in 6 well plate and incubated for 24 h at 37˚C, 5% CO_2. After 24 h, cells were treated with different concentrations of GW843682X ranging from 0 to15 µM, DMSO was used as a control. Next, cells were plated at the density of 1000/ well in 6 well plates. After 7-10 days, the colonies were fixed with 10% formaldehyde and stained with 0.05% crystal violet. The survival fraction upon the GW843682X treatment was calculated.

PC3 xenograft tumor cell recovery and subline generation

Xenograft tumors were established as previously described [8]. Briefly, 1x10^6 PC3 Luc2/RGB cells were injected subcutaneously in 8 to 12 weeks old male immunodeficient NSG mice (NOD.Cg-Prkdcscid Il2rgtm1Wjl/SzJ; The Jackson Laboratory, Stock 005557). The animal experiments were approved by the local animal experiment approval committee (Behörde für Justiz und Verbraucherschutz - Lebensmittelsicherheit und Veterinärwesen, assigned project No. N017/2020). The primary tumors were surgically removed before reaching a volume of 1 cm^3 or ulcerated the mouse skin to investigate the outgrowth capacity of spontaneous metastases after prolonged growth periods. The mice were sacrificed when the relapsing tumor reached a volume of ~1 cm^3. At necropsy, tissue of the relapsing tumor and the lung mechanically dissociated using a scalpel and filtered through a 70 µm cell strainer using a syringe plunger. The bones of the hind limbs were cut transversally in the middle of the diaphysis, and the bone marrow was harvested by centrifuging for 30 sec on 5500 g (after placing the bones with the opened sides down in PCR tubes placed in 1.5 ml Eppendorf tubes). The cell suspensions of different tissues were resuspended in a culture medium

(RPMI-1640 medium supplemented with 10% FBS, 100 U/ml penicillin, 100 µg/ml streptomycin, and 0.5 µg/ml puromycin). Expansion of adherent tumor cell cultures was monitored in a light microscope, and sub-cultivation of xenograft tumor, lung metastasis, and bone marrow metastasis sublines was conducted as appropriate. These sublines were then re-injected subcutaneously into novel NSG mice, developing primary tumors surgically removed, and tumor cells recovered from relapse tumors, lung metastases, or bone metastases as appropriate. The entire procedure was repeated four times in total, and the 1st and 4th generation sublines from the primary tumor, lung metastasis, and bone metastasis were compared in this study.

Syngeneic immunocompetent tumor model

Intracardiac injection of murine prostate cancer cells RM1(BM) with or without the knockdown of Aldh1a1 and Aldh1a3 gene followed by immunofluorescence analysis was used to examine the ability of the cells to home to the bone. Anesthetized animals were placed in a supine position for tumor cell injection. Male C57BL/6 mice (8-9 weeks old) were injected into the left ventricle with shNS (control), shAldh1a1, or shAldh1a3 cells (1×10^5 cells per mouse). On day 3 post injections, mice were terminated by cervical dislocation, the legs from the mice were removed, and knees detached from the femur and tibia. Then, the bones were fixed in 4% PFA overnight at 4°C. The following day, bones were washed in PBS and dried. Decalcification was performed with 2 ml of Osteosoft reagent (Merck Millipore) at 37°C for 5 days. Afterward, the bones were embedded in OCT Tissue-Tek specimen matrix compound (Sakura) and cut into 10 µm thick sections with Cryotome. The bones were stored at -20°C. Experiments were approved by the Landesdirektion Sachsen.

Immunofluorescence of bone metastasis on frozen sections

First, frozen sections were left at room temperature for at least 30 min and the area of the section was marked with Pap-Pen (Thermo Fisher Scientific). After 5 min fixation of sections with 4% PFA, they were washed and permeabilized with Triton-X-100 0.01% in PBS. After 3 times washes with PBS, the sections were blocked with Protein block Serum-free DAKO (Agilent) for 30 to 45 min at RT. Then, incubation with primary antibodies was done in DAKO Antibody diluent with background reducing components (Agilent) at 4°C overnight. The next day, after 3 times washes with PBS, the sections were incubated with a secondary antibody in DAKO Antibody diluent for 1.5 h at RT. Afterward, sections were washed 3 times with PBS and stained with DAPI (1 mg/ml, 1:5000 in 1xPBS) for 5 min at room temperature. Later, the slides were mounted with fluorescent mounting medium DAKO (Agilent) and left at 4°C overnight. The imaging was performed with WF Slide scanner Axioscan (Zeiss). The antibodies used in the study are listed in Supplementary Table 3.

RNA sequencing analysis

RNAseq dataset was prepared using SUMO software as follows. Genes expressed at the noise level were filtered out, and remaining data were quantile normalized across all samples. Further, gene expression values were median normalised and log2 transformed. Significantly down-regulated genes predicted as off-targets by DSIR web tool (http://biodev.cea.fr/DSIR/DSIR.html) were filtered out, and remaining deregulated genes were evaluated for enrichment or depletion between different treatments comparing to randomly expected numbers.

Analysis of the patient cohort data

The publicly available datasets (TCGA PRAD, MSKCC and Metastatic PCa SU2C/PCF Dream Team [9]) were downloaded from cBioportal https://www.cbioportal.org/ and analyzed using SUMO software https://angiogenesis.dkfz.de/oncoexpress/software/sumo/. For evaluation of correlation with ALDH1A1 and ALDH1A3 the RT2 profiler PCR Array gene sets (Qiagen) were used, the Pearson correlation coefficients for all genes included in each gene set were determined, and the median correlation coefficients were calculated. For Kaplan-Meier survival analysis, the biochemical recurrence-free survival time was determined based on provided "Days to PSA" and "Days to biochemical recurrence first" data, and the patient groups were defined by the optimal cut-off scan procedure.

Supplementary Tables and Figures

Table S1. Clinicopathological characteristics of PCa patients whose tumor samples were used for immunohistochemical analysis of ALDH1A1 and ALDH1A3 expression (n = 215).

Table S2. shRNA constructs used for knockdown of Aldh1a1 and Aldh1a3.

Table S3. Antibodies, primers, and siRNA oligonucleotides used for the study.

Table S4. Gene sets used for the correlative analysis.

Table S1. Clinicopathological characteristics of PCa patients whose tumor samples were used for immunohistochemical analysis of ALDH1A1 and ALDH1A3 expression (n = 215).

Parameter	n	%
**WHO Grade Group**		
1	74	34.4%
2	79	36.7%
3	31	14.4%
4	17	7.9%
5	14	6.5%
**T-Stage**		
T2	125	58.1%
T3a	38	17.7%
T3b	33	15.3%
T4	2	0.9%
missing	17	7.9%
**N-Status**		
N0	191	88.8%
N1	20	9.3%
missing	4	1.9%
**R-Status**		
R0	142	66.0%
R1	62	28.8%
missing	11	5.1%
**Biochemical recurrence**		
Yes	143	66.5%
No	72	33.5%
**Death**		
Yes	206	95.8%
No	7	3.3%
missing	2	0.9%
**Follow-up (month)** (max;min;median)	160.53; 0; 50.5	

Table S 2. shRNA constructs used for knockdown of Aldh1a1 and Aldh1a3.

Gene	Name	Vector	Hairpin sequence
Non-silencing	shNS	pLKO.1	CCTAAGGTTAAGTCGCCCTCGCTC GAGCGAGGGCGACTTAACCTTAGG
Aldh1a1	shAldh1a1	pLKO.1	CCCAGTTCTTATCCAAGAATACTC GAGTATTCTTCGGATAAGAACTGGG
Aldh1a3	shAldh1a3	pLKO.1	CGAATCCAAGAGTGGAAGAAACTC GAGTTTCTTCCACTCTTGGATTCG

Table S3. Antibodies, primers, and siRNA oligonucleotides used for the study.

siRNA oligonucleotides used for knockdown of gene expression

**Gene**	**siRNA name**	**Sequence**
Scrambled siRNA	siSCR	ss GCAGCUAUAUGAAUGUUGUdTdT<br>as ACAACAUUCAUAUAGCUGCdTdT
ALDH1A1	siALDH1A1 #1	ss UCACAUGGAUAUAGACAAAdTdT<br>as UUUGUCUAUAUCCAUGUGAdTdT
ALDH1A1	siALDH1A1 #2	ss GAUCCAGGGCCGUACAAUAdTdT<br>as UAUUGUACGGCCCUGGAUCdTdT
ALDH1A3	siALDH1A3 #1	ss AGGAAAUGGCAGAGAACUAdTdT<br>as UAGUUCUCUGCCAUUUCCUdTdT
ALDH1A3	siALDH1A3 #2	ss UCGUGGAGGAGCAGGUCUAdTdT<br>as UAGACCUGCUCCUCCACGAdTdT
Scrambled<br>siRNA pool	siSCR	ss GCAGCUAUAUGAAUGUUGUdTdT<br>as ACAACAUUCAUAUAGCUGCdTdT<br>ss UGCGCUAGGCCUCGGUUGCdTdT<br>as GCAACCGAGGCCUAGCGCAdTdT
AR	siAR	ss CCAAAGGGCUAGAAGGCGAdTdT<br>as UCGCCUUCUAGCCCUUUGGdTdT<br>ss AUUGAUAAAUUCCGAAGGAdTdT<br>as UCCUUCGGAAUUUAUCAAUdTdT
CTNNB1	siCTNNB1	ss GGGUACGAGCUGCUAUGUUdTdT<br>as AACAUAGCAGCUCGUACCCdTdC<br>ss GGUGGUGGUUAAUAAGGCUdTdT<br>as AGCCUUAUUAACCACCACCdTdG
RARG	siRARG	ss GGAGAACCCUGAAAUGUUUdTdT<br>as AAACAUUUCAGGGUUCUCCdTdT<br>ss GGGACCCUCCUACACUACAdTdT<br>as UGUAGUGUAGGAGGGUCCCdTdT<br>ss GAGAUGGAUGACACCGAGAdTdT<br>as UCUCGGUGUCAUCCAUCUCdTdT<br>ss GAGGAAGCCUCUAUUUAUUdTdT<br>as AAUAAAUAGAGGCUUCCUCdTdT
RARA	siRARA	ss CUGUGAGAAACGACCGAAAdTdT<br>as UUUCGGUCGUUUCUCACAGdTdT<br>ss CUGCGAAGCAUCAGCGCCAdTdT<br>as UGGCGCUGAUGCUUCGCAGdTdT<br>ss UAAAGGUCUACGUGCGGAAdTdT<br>as UUCCGCACGUAGACCUUUAdTdT<br>ss CGGAUCUGCACGCGGUACAdTdT<br>as UGUACCGCGUGCAGAUCCGdTdT
RXRA	siRXRA	ss GGCAAGGACCGGAACGAGAdTdT<br>as UCUCGUUCCGGUCCUUGCCdTdT<br>ss CGAACGACCCUGUCACCAAdTdT<br>as UUGGUGACAGGGUCGUUCGdTdT<br>ss UGACGGAGCUUGUGUCCAAdTdT<br>as UUGGACACAAGCUCCGUCAdTdT<br>ss CAGCCGGGAAGGUUCGCUAdTdT

		as UAGCGAACCUUCCCGGCUGdTdT
PLK3	siPLK3	ss AGAAAGACUGUGCACUACAdTdT<br>as UGUAGUGCACAGUCUUUCUdTdT<br>ss CAGCGCGAGAAGAUCCUAAdTdT<br>as UUAGGAUCUUCUCGCGCUGdTdT<br>ss CCCGAUCGACUCCCUAUCAdTdT<br>as UGAUAGGGAGUCGAUCGGGdTdT<br>ss GCGGCAGCGACUCCGCUAUdTdT<br>as AUAGCGGAGUCGCUGCCGCdTdT

Primers used for RT-qPCR

Gene	Sequence (5’→3’)
ACTB	F 5'- ATGGAGTCCTGTGGCATCCA-3'<br>R 5'- AGTACTTGCGCTCAGGAGGA-3'
RPLP0	F 5'- CTCAACATCTCCCCCTTCTCCTT-3'<br>R 5'- TGATGCAACAGTTGGGTAGCC-3'
HPRT1	F 5'-CTTTGCTGACCTGCTGGATTAC-3'<br>R 5'- TTGCGACCTTGACCATCTTTG-3'
ALDH1A1	F 5'- GAATGGCATGATTCAGTGAGTGG-3'<br>R 5'- CAGCCAACTTGTATAATAGTCG-3'
ALDH1A2	F 5'- TTGCAGGGCGTCATCAAAAC-3'<br>R 5'- ACACTCCAATGGGTTCATGTC-3'
ALDH1A3	F 5'- TCTCGACAAAGCCCTGAAG-3'<br>R 5'- TATTCGGCCAAAGCGTATTC-3'
ALDH1B1	F 5'- CCATGTGGACAGAGCTGGGAG-3'<br>R 5'- CTGCTGCCGAGGAGTAGC-3'
ALDH3A1	F 5'- GCAGACCTGCACAAGAATGA-3'<br>R 5'- TGTAGAGCTCGTCCTGCTGA-3'
ALDH3A2	F 5'- TGCACTTCACGCTCAACTCT-3'<br>R 5'- GACTGGCTGTTGGGAGGATA-3'
ALDH3B1	F 5'- GCCAGGCTGATCTTGAACTC-3'<br>R 5'- ACAGAGAAGGTCCTGGCTGA-3'
ALDH5A1	F 5'- ACCAATTCTTGGTGCAAAGG-3'<br>R 5'- GTTGGTGTCGTTTTCCACCT-3'
ALDH2	F 5'- ATGGCAAGCCCTATGTCATCT-3'<br>R 5'CCGTGGTACTTATCAGCCCA-3'
AR	F 5'- CATCTTGTCGTCTTCGGAAATGTTA-3'<br>R 5'- GAAGCCTCTCCTTCCTCCTGTAGTT-3'
BRCA1	F 5'- CATCATTCACCCTTGGCACA-3'<br>R 5'- GGGGTATCAGGTAGGTGTCC-3'
CCN1	F 5' - GTTGGAAAAGGCAGCTCAC-3'<br>R 5'- CATTTCTTGCCCTTTTTCAG-3'
CTNNB1	F 5'- ATTTGATGGAGTTGGACATGGC-3'<br>R 5'- CCAGCTACTTGTTCTTGAGTGAAGG-3'
MMP11	F 5'- GGACCTCACCTACAGGATC-3'<br>R 5'- GTCCCCATGCCAGTACCTG-3'
RARA	F 5'- CAAGTGCATCATTAAGACTGTGG-3'<br>R 5'- CGAGAAGGTCATGGTGTCC-3'
RARG	F 5'- GCATGTCCAAGGAAGCTGTGC-3'<br>R 5'- CTGCACTGGAGTTCGTGGT-3'
RXRA	F 5'- GCTGCACGTCCACCGGAAC-3'<br>R 5'- CCTTGGAGTCAGGGTTAAAGAGG-3'
PLK3	F 5'- CCTCAACTACTTGCACCAG-3'<br>R 5'- CAGAGGAGCGTGTACATGAC-3'
SLC7A8	F 5'- CCACATTTGGAGGAGTTAATGG-3'

	R 5'- GTACATGTCGCTGGTGACC-3'
SNAI2	F 5'- TCGGACCCACACATTACCTT-3'
	R 5'- TGAGCCCTCAGATTTGACCT-3'
Mouse/rat Gapdh	F 5'- TTCAACGGCACAGTCAAGG-3'
	R 5'- ACATACTCAGCACCAGCATCAC-3'
Mouse Aldh1a1	F 5'- ATACTTGTCGGATTTAGGAGGCT-3'
	R 5'- GGGCCTATCTTCCAAATGAACA-3'
Mouse Aldh1a3	F 5'- GGGTCACACTGGAGCTAGGA-3'
	R 5'- CTGGCCTCTTCTTGGCGAA-3'

Primers used for ChIP-qPCR

Gene	Sequence (5'→3')
prKLK3	F 5'- GCAAAGGATCTAGGCACGTGAG-3'
	R 5'- CACCCAGAGCTGTGGAAGG-3'
prRIG-1	F 5'- GCAGCTGTACTTATACCCAC-3'
	R 5'- CTAGCAGAGGGTGATGGAAG-3'
prPLK3 #1	F 5'- GGGTATTTTCAGAGGAAGGG-3'
	R 5'- CCAGCAGCGGAGAGGCAG-3'
prPLK3 #2	F 5'- CTGCATGGTGACTAGCTG-3'
	R 5'- CCAGCGTTTTATCAATGAAC-3'

Antibody	Dilution and host	Vendor and catalogue number
**Antibodies used for Immunofluorescence**		
γH2A.X (Ser139)	1:1000, Mouse	Sigma-Aldrich, 05-636
Endomucin	1:200, Goat	Thermo Fisher Scientific, #PA5-47648
GFP Tag	1:200, Rabbit	Thermo Fisher Scientific, #A6455
Anti-Goat IgG (Alexa Fluor 555)	1:350, Donkey	Thermo Fisher Scientific, #A-21432
Anti-Rabbit IgG (Alexa Fluor 488)	1:350, Donkey	Thermo Fisher Scientific, #A32731
**Antibodies used for Immunohistochemical staining**		
anti-ALDH1A1	1:50, Rabbit	Thermo Fisher Scientific, #PA5-11537
anti-ALDH1A3	1:50, Rabbit	Atlas Antibodies, HPA046271
**Antibodies used for Western Blot**		
Chk1	1:1000, Mouse	Cell Signaling Technology, #2360
pChk1 (Ser296)	1:1000, Rabbit	Cell Signaling Technology, #2349
Chk2	1:1000, Rabbit	Cell Signaling Technology, #6334
pChk2 (Thr68)	1:1000, Rabbit	Cell Signaling Technology, #2197
ALDH1A1	1:1000, Mouse	Santa Cruz Biotechnology, sc-374076
ALDH1A3	1:1000, Rabbit	Atlas Antibodies, HPA046271
p21	1:1000, Rabbit	Cell Signaling Technology, #2347
γH2A.X (Ser139)	1:1000, Mouse	Sigma-Aldrich, 05-636
AR	1:1000, Rabbit	Cell Signaling Technology, #5153
EpCAM	1:1000, Mouse	Santa Cruz Biotechnology, sc-21792
SNAI1	1:1000, Mouse	Santa Cruz Biotechnology, sc-271977
c-MYC	1:1000, Rabbit	Cell Signaling Technology, #5605
GAPDH	1:1000, Rabbit	Santa Cruz Biotechnology, sc-25778
PLK3	1:1000, Rabbit	Cell Signaling Technology, #4896
**Antibodies used for ChIP**		
AR	2μg, Rabbit	Cell Signaling Technology, #5153
RARA	2μg, Rabbit	Cell Signaling Technology, #62294
IgG	2μg, Rabbit	Cell Signaling Technology, #3900

Table S4. Gene sets used for the correlative analysis.

Androgen Receptor Signaling Targets:

SORD	PGC	ERRFI1	TIPARP	FZD5	IGF1R	GUCY1A3	FOS	HERC3	ZNF189	NKX3-1	NFKB2
HPGD	DBI	NFKBIA	CITED2	APPBP2	CYP2U1	ABHD2	MAF	MYC	SNAI2	ADAMTS1	SP1
ORM2	ORM1	ELL2	KLK3	KLK2	RAB4A	ELK1	PAK1IP1	SLC26A2	SPDEF	SMS	CENPN
REL	SEC22C	SGK1	ACKR3	NDRG1	TRIB1	KLK4	IGFBP5	TSC22D1	DHCR24	NFKB1	RELA
VAPA	MME	PIAS1	SRF	PIK3R3	LRIG1	LRRFIP2	RHOU	EAF2	WIPI1	ENDOD1	STEAP4
ZBTB10	ABCC4	PMEPA1	MT2A	SLC45A3	LIFR	STK39	IRS2	NCAPD3	FAM105A	TMPRSS2	ACSL3
MAP7D1	JUN	CAMKK2	VIPR1	PPAP2A	ZBTB16	TPD52	TSC22D3	AR	ALDH1A3	FKBP5	KRT8

WNT Signaling Targets:

FGF9	GDF5	FGF4	TGFB3	MMP7	SOX9	ANGPTL4	FST	NRCAM	DKK1	SIX1	TWIST1
BMP4	VEGFA	GJA1	PDGFRA	PITX2	RUNX2	LEF1	NRP1	MET	EFNB1	T	CTGF
MYC	TCF4	IL6	NANOG	SFRP2	PLAUR	SOX2	WISP2	WNT3A	FOSL1	MMP9	POU5F1
IGF2	IRS1	WISP1	SMO	CDKN2A	BIRC5	GDNF	JAG1	MMP2	TCF7	ID2	CDH1
IGF1	CD44	TLE1	CDON	FN1	PPAP2B	NTRK2	DAB2	AXIN2	CCND1	EGR1	DLK1
KLF5	BTRC	ABCB1	WNT9A	DPP10	PTCH1	TCF7L2	ANTXR1	LRP1	ETS2	CUBN	WNT5A
PTGS2	PPARD	FZD7	TCF7L1	CCND2	AHR	CACNA2D3	EGFR	FGF7	FGF20	CEBPD	BGLAP

Extracellular Matrix and Adhesion Molecules:

SPP1	MMP12	MMP7	VTN	MMP10	MMP14	MMP13	ITGB5	HAS1	SELP	MMP15	ECM1
SELL	MMP1	SELE	COL12A1	TIMP2	VCAM1	SPARC	ADAMTS13	MMP11	TNC	ITGAV	ITGAL
CTGF	COL6A2	ADAMTS1	THBS1	LAMC1	THBS3	ITGB2	COL6A1	TIMP3	COL7A1	SGCE	TGFBI
ITGA2	MMP3	MMP9	CTNNA1	CNTN1	COL5A1	MMP8	ITGA5	CLEC3B	MMP2	THBS2	COL16A1
ITGA4	CDH1	LAMA3	COL4A2	ICAM1	LAMA1	ITGA3	CD44	CTNND1	FN1	COL1A1	ITGB4
NCAM1	LAMA2	TIMP1	ITGB1	CTNNB1	ADAMTS8	LAMB1	ITGA7	COL11A1	VCAN	ITGA6	COL15A1
ITGAM	SPG7	LAMB3	CTNND2	COL14A1	ITGA1	ITGA8	COL8A1	ITGB3	PECAM1	MMP16	KAL1

Epithelial to Mesenchymal Transition:

SPP1	TGFB1	BMP1	FGFBP1	TGFB3	ILK	SMAD2	ZEB2	SNAI1	STEAP1	TWIST1	BMP2
RGS2	IL1RN	GNG11	MSN	DSC2	WNT11	SPARC	TMEM132A	ERBB3	ZEB1	GSC	ITGAV
BMP7	MST1R	TCF3	TCF4	SNAI2	COL5A2	ESR1	TGFB2	MMP3	MAP1B	MMP9	OCLN
FOXC2	PDGFRB	CAV2	AKT1	KRT14	ITGA5	JAG1	MMP2	TFPI2	GEMIN2	CDH1	COL3A1
PTK2	CAMK2N1	VIM	FN1	KRT19	GSK3B	DESI1	STAT3	CDH2	TIMP1	SNAI3	ITGB1
SERPINE1	CTNNB1	NODAL	PLEK2	AHNAK	VCAN	IGFBP4	TMEFF1	TSPAN13	NOTCH1	RAC1	SOX10
WNT5A	NUDT13	COL1A2	CALD1	FZD7	WNT5B	DSP	EGFR	PTP4A1	F11R	VPS13A	KRT7

Angiogenesis:

CXCL1	IFNG	TGFB1	ANPEP	FGFR3	MMP14	CXCL8	ANGPTL4	FIGF	IFNA1	HPSE	CCL11
F3	SPHK1	VEGFA	CDH5	ENG	CXCL9	VEGFB	TIE1	PF4	MDK	TEK	CXCL5
TIMP2	IL1B	NRP1	EFNB2	ID1	CXCL10	ITGAV	PLG	S1PR1	CTGF	FLT1	KDR
PDGFA	IL6	HIF1A	THBS1	COL18A1	PLAU	CXCL6	PGF	TNF	VEGFC	SERPINF1	TIMP3
TGFB2	MMP9	EFNA1	AKT1	JAG1	MMP2	THBS2	EPHB4	IGF1	EGF	HGF	EDN1
LECT1	FN1	CCL2	TGFA	TIMP1	NRP2	NOTCH4	SERPINE1	ADGRB1	FGF2	PROK2	TGFBR1
COL4A3	ANG	NOS3	PTGS1	TYMP	ERBB2	LEP	ANGPT1	ANGPT2	FGF1	ITGB3	PECAM1

Osteogenesis:

SPP1	BGN	TGFB1	BMP1	CSF2	TGFB3	GDF10	MMP10	SOX9	COMP	CSF3	SMAD2
IGF1R	SMAD3	TWIST1	BMP4	VEGFA	BMP2	ALPL	SMAD4	VEGFB	TGFBR2	DLX5	VCAM1
CDH11	CD36	BMP3	RUNX2	ACVR1	BMP7	CSF1	ANXA5	COL2A1	IHH	FLT1	PDGFA
PHEX	SERPINH1	TNF	ITGA2	TGFB2	MMP9	CALCR	BMPR2	FGFR2	CHRD	IGF2	COL5A1
MMP8	MMP2	BMPR1B	NOG	ICAM1	COL3A1	IGF1	EGF	ITGA3	NFKB1	BMP5	FN1
COL1A1	CTSK	FGFR1	VDR	ITGB1	BMP6	FGF2	TGFBR1	AHSG	COL10A1	SMAD1	COL15A1
ITGAM	SP7	SMAD5	COL1A2	COL14A1	ITGA1	GLI1	EGFR	FGF1	TNFSF11	BMPR1A	BGLAP

Tumor Metastasis:

CXCR2	TGFB1	MMP7	MMP10	MMP13	NME4	SMAD2	DENR	HRAS	HPSE	VEGFA	SMAD4
GNRH1	IL18	HTATIP2	KISS1	BRMS1	TIMP2	MGAT5	CDH11	NME1	IL1B	MET	MMP11
TSHR	CHD4	MYC	CCL7	CD82	MYCL	FLT4	FXYD5	TP53	CTSL	TNFSF10	PLAUR
CXCR4	SRC	ETV4	PNN	TIMP3	MMP3	RPSA	MMP9	CTNNA1	CDKN2A	MDM2	CTBP1
CST7	MMP2	APC	TRPM1	CDH1	COL4A2	IGF1	CD44	NR4A3	MCAM	HGF	RORB
FN1	MTA1	CTSK	SSTR2	PTEN	KISS1R	SERPINE1	METAP2	KRAS	SYK	ITGA7	EWSR1
TCF20	MTSS1	TIMP4	EPHB2	CXCL12	RB1	FGFR4	NF2	SET	ITGB3	CDH6	FAT1

DNA Damage Signaling Pathway:

CDC25C	CDC25A	LIG1	GADD45G	RAD17	CHEK1	MBD4	TOPBP1	BARD1	NTHL1	APEX1	PNKP
GADD45A	SUMO1	RAD21	CSNK2A2	XRCC1	PMS1	XRCC2	PCNA	CRY1	HUS1	UNG	PMS2

XPC	ABL1	BLM	PRKDC	TP53	BAX	RAD51	FEN1	SMC1A	BBC3	XRCC3	MSH2
ATM	RAD50	FANCA	FANCG	NBN	CIB1	MAPK12	RAD1	OGG1	DDB1	PPP1R15A	ERCC1
PARP1	MPG	XPA	DDIT3	TP53BP1	PPM1D	MCPH1	MDC1	FANCD2	MRE11A	RAD9A	ATRX
TP73	RAD18	ERCC2	ATRIP	RBBP8	RPA1	MLH1	RNF168	ATR	BRCA1	REV1	H2AFX
BRIP1	DDB2	XRCC6	EXO1	CHEK2	MLH3	RNF8	RAD51B	CDK7	MSH3	SIRT1	CDKN1A

Retinoic Acid Signaling:

ISL1	RARG	CDX1	EPO	SOX9	RARRES3	RXRA	CHD7	STRA6	RXRG	DCX	HOXA5
RBP4	FGF8	HOXA1	NEUROD1	BMP2	ALDH1A1	MSX2	CYP26B1	PITX2	DLX5	HOXB1	PPARA
SHH	EFNB1	RXRB	FABP5	ADH1A	MYC	UCP1	WNT8A	STRA8	PPARG	PAX6	TFAP2C
NANOG	CRABP2	HOXB4	SOX2	HNF1B	KLF4	TGFB2	BHLHE40	FOXG1	LHX1	FOXA1	RBP2
JAG1	CRABP1	DHRS3	LRAT	ALDH1A2	RET	RBP1	TGM2	CD38	MAFB	EGR1	CYP1B1
OLIG2	HSD17B2	OTX2	RDH10	DHRS9	TUBB3	RARA	CYP26A1	LEFTY1	NRIP1	APOA2	PLAT
ASCL1	GBX2	MEIS2	RARB	GATA4	WNT5A	PPARD	SREBF1	GLI1	ALDH1A3	CYP26C1	TBX1

Cancer Stem Cells:

EPCAM	KITLG	CXCL8	LIN28B	CHEK1	MYCN	ZEB2	ETFA	DKK1	SNAI1	TWIST1	ALDH1A1
ENG	LIN28A	HDAC1	ZEB1	DACH1	THY1	ID1	FOXA2	BMP7	MYC	DDR1	NOS2
MS4A1	AXL	NANOG	TWIST2	PLAUR	SOX2	WNT1	KLF17	KLF4	MUC1	ITGA2	PROM1
FGFR2	POU5F1	ATM	SMO	DLL1	CD34	JAG1	WWC1	ITGA4	TAZ	FLOT2	EGF
CD44	NFKB1	MAML1	ALCAM	CD38	GSK3B	STAT3	SAV1	ITGB1	ABCG2	PTPRC	FOXP1
LATS1	PLAT	DLL4	TGFBR1	ITGA6	KIT	MERTK	PTCH1	NOTCH2	YAP1	NOTCH1	ERBB2
WEE1	DNMT1	JAK2	ABCB5	IKBKB	FZD7	ATXN1	GATA3	SIRT1	PECAM1	BMI1	CD24

DNA Repair:

LIG1	CCNO	SMUG1	BRCA2	XRCC6BP1	NTHL1	APEX1	PNKP	RAD21	POLD3	XRCC1	LIG3
PMS1	RAD51D	XRCC2	CCNH	APEX2	ERCC5	UNG	PMS2	XPC	RAD52	PRKDC	RAD51
FEN1	RAD54L	MSH6	XAB2	DMC1	TOP3A	XRCC3	MSH2	ATM	RAD50	RAD51C	MSH5
OGG1	DDB1	TOP3B	MGMT	ERCC1	POLB	PARP1	MPG	XPA	PARP2	XRCC5	SLK
MRE11A	ERCC4	RAD23B	RAD23A	POLL	RPA3	LIG4	NEIL1	XRCC4	RAD18	ERCC2	MUTYH
RPA1	RFC1	MLH1	TDG	ERCC8	ATR	BRCA1	MMS19	NEIL2	BRIP1	DDB2	ERCC3
XRCC6	ATXN3	EXO1	NEIL3	MLH3	MSH4	PARP3	RAD51B	ERCC6	CDK7	MSH3	TREX1

Supplementary Figure 1

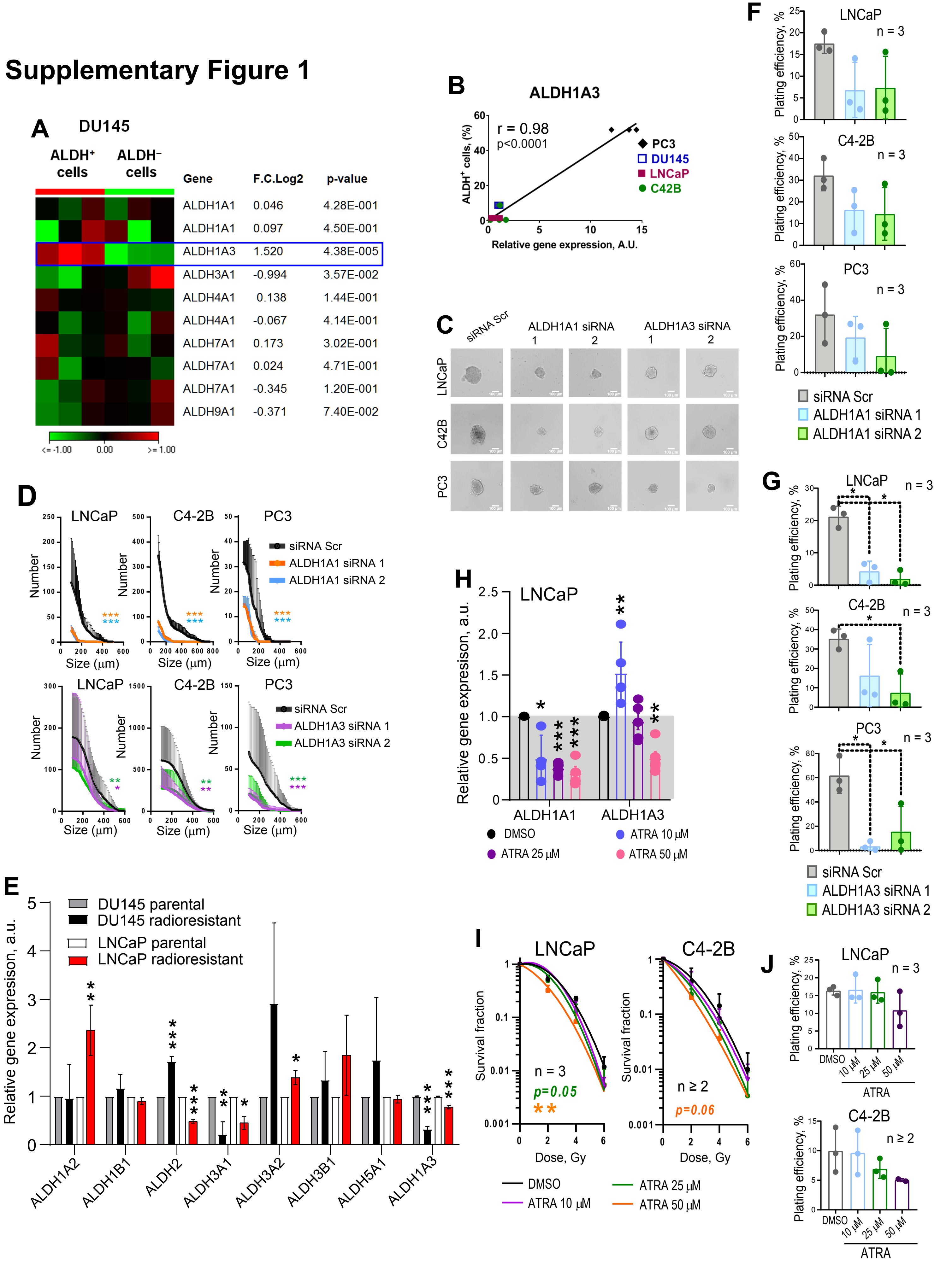

Supplementary Figure 1. ALDH1A1 and ALDH1A3 as regulators of radiosensitivity. (**A**) Levels of ALDH genes by gene expression profiling of $ALDH^+$ and $ALDH^-$ cell populations isolated by FACS from DU145 PCa cells as we described previously [7]. (**B**) Correlation of ALDH1A3 expression with a mean size of $ALDH^+$ population in four prostate cancer cell lines. (**C**) Representative images of prostatospheres are shown for cells transfected with scrambled siRNA (control) and with ALDH1A1 or ALDH1A3 siRNAs. Scale bar = 100μm. (**D**) Complementary cumulative distribution for the number and size of tumor spheres after ALDH1A1 or ALDH1A3 depletion. The graph represents the number of spheres larger than thresholds. N≥3; Error bars = SEM; *p < 0.05; **p < 0.01; ***p < 0.001. (**E**) Quantitative real-time PCR (RT-qPCR) analysis of ALDH gene expression in DU145 and LNCaP parental and radioresistant cell lines. N = 3; Error bars = SD; *p < 0.05; **p<0.01; ***p<0.001. (**F**) Plating efficiency of prostate cancer cells after transfection with scrambled (Scr) siRNA or ALDH1A1 siRNAs. Error bars = SD. (**G**) Plating efficiency of prostate cancer cells after transfection with scrambled (Scr) siRNA or ALDH1A3 siRNAs. Error bars = SD. *p < 0.05. (**H**) Relative mRNA expression of ALDH1A1 and ALDH1A3 upon all-trans retinoic acid (ATRA) treatment. Data are plotted relative to the DMSO control sample. N ≥ 3; Error bars = SD. *p < 0.05; **p < 0.01; ***p < .001. (**I**) 2D radiobiological colony forming assay after cell pre-treatment with ATRA at the indicated concentration for 48 h. Cells treated with DMSO were used as control. Error bars = SD; **p < 0.01. (**J**) Plating efficiency of prostate cancer cells after cell pre-treatment with ATRA at the indicated concentration for 48 h. Cells treated with DMSO were used as control. Error bars = SD.

Supplementary Figure 2

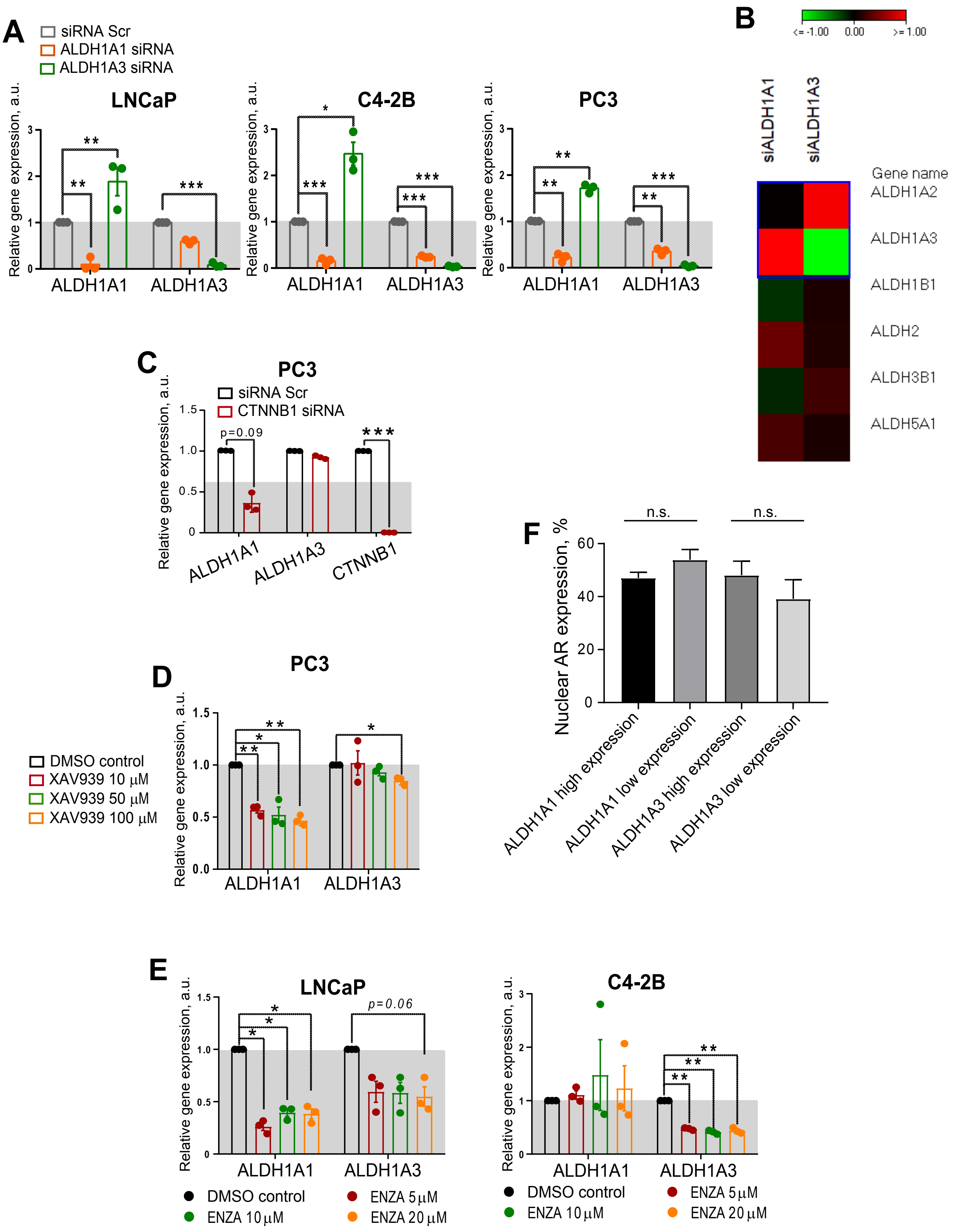

Supplementary Figure 2. ALDH1A1 and ALDH1A3 are interconnected with AR and β-catenin signaling pathways. (**A**) qPCR analysis of ALDH1A1 and ALDH1A3 expression after reciprocal knockdown. Data are shown relative to the control siSCR sample. N = 3; Error bars = SD. *p < 0.05; **p < 0.01; ***p < 0.001. (**B**) RNAseq analysis of the members of the ALDH family contributing to Aldefluor activity showed that only ALDH1A2 was highly upregulated after ALDH1A3 knockdown. (**C**) RT-qPCR analysis of the ALDH1A1 and ALDH1A3 expression after CTNNB1 knockdown in PC3 cells. Data are plotted relative to the Scr siRNA. N ≥ 3; Error bars = SD; ***p < 0.001. (**D**) Analysis of ALDH1A1 and ALDH1A3 genes expression upon inhibition of WNT signaling pathway with XAV939 inhibitor. Normalized to housekeeping gene RPLP0 and plotted relative to the DMSO control sample. The cells were serum-starved in DMEM medium with 3% FBS for 24 h, followed by treatment with XAV939 at different concentrations. N = 3; Error bars = SD. *p < 0.05; **p<0.01. (**E**) Analysis of ALDH1A1 and ALDH1A3 genes expression upon inhibition of AR signaling with enzalutamide. Normalized to housekeeping gene RPLP0 and plotted relative to the DMSO control sample. The cells were serum-starved in RPMI medium with 3% FBS for 24 h followed by treatment with Enzalutamide at different concentrations. N = 3; Error bars = SEM. *p < 0.05; **p < 0.01. (**F**) Analysis of the nuclear AR expression in patients with PCa with different levels of ALDH1A1 and ALDH1A3 protein expression in tumor tissues (Lübeck cohort). Error bars = SEM; n.s. – non-significant.

Supplementary Figure 3

A

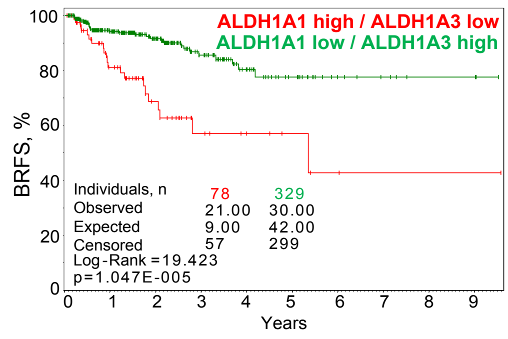

B

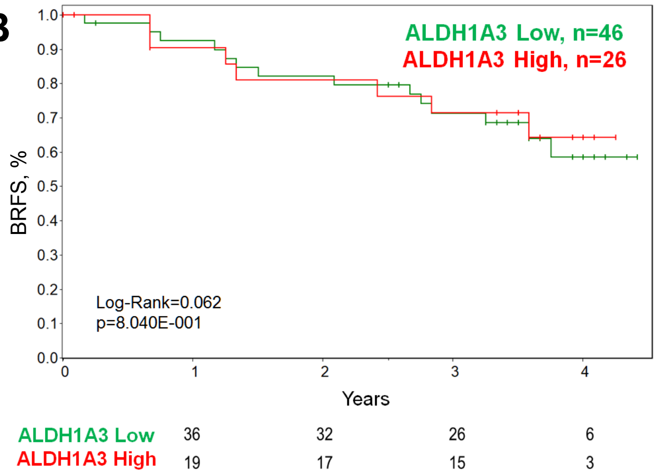

C

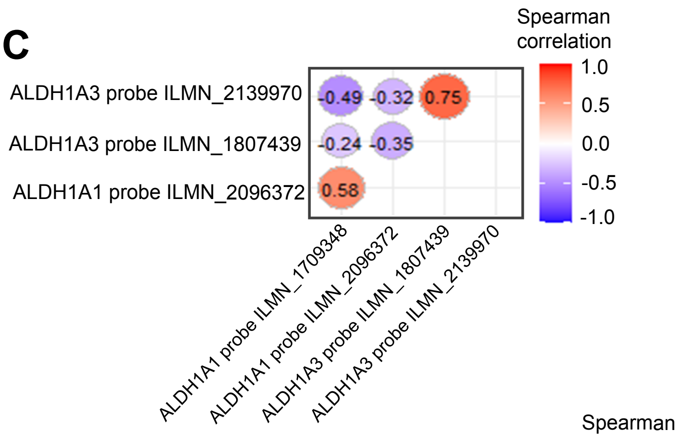

D

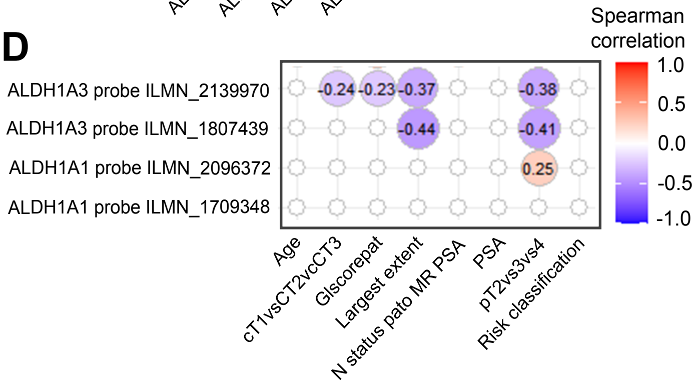

E

Pearson Chi-square p = 0.007

ALDH1A1 expression in tumors (RPE)	N0	N1
Negative	126 (66%)	11 (55%)
Positive in single cells	38 (19.9%)	3 (15%)
Low expression	15 (7.9%)	2 (10%)
Moderate expression	7 (3.7%)	0
High expression	5 (2.6%)	4 (20%)

G

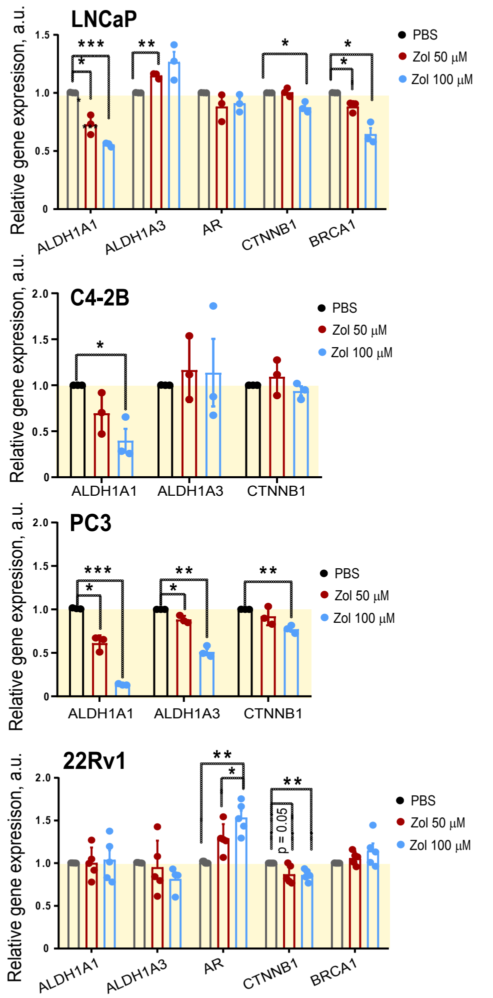

F ALDH1A3

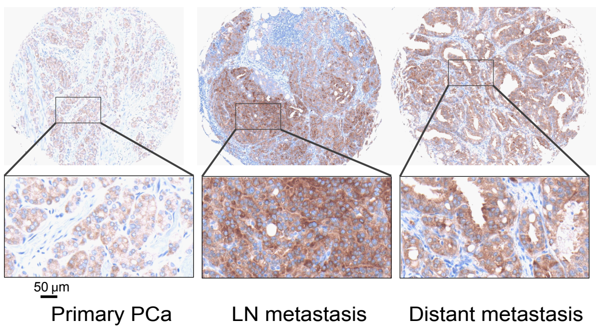

H

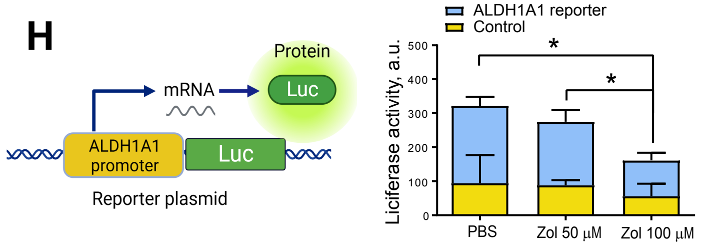

Supplementary Figure 3. The levels of ALDH1A1 and ALDH1A3 expression are clinically relevant. (**A**) Analysis of the biochemical recurrence-free survival (BRFS) of TCGA PRAD patients' cohort stratified based on a signature combining ALDH1A1-high with ALDH1A3-low expression. (**B**) The Kaplan-Meier analysis of biochemical recurrence-free survival of patients with low (green) compared to high (red) ALDH1A3 expression levels (Lübeck cohort). N = 72. (**C**) Analysis of the gene expression dataset for patients with primary intermediate or high-risk PCa (Oslo cohort [10], n = 95) confirmed a negative correlation between ALDH1A1 and ALDH1A3 genes. (**D**) ALDH1A3 negatively correlates with clinical parameters associated with cancer aggressiveness (Oslo cohort [10], n = 95). (**E**) ALDH1A1 expression on primary tumors is associated with positive nodal status (Lübeck cohort). N= 211; RPE – relative protein expression. (**F**) Representative images showing ALDH1A3 expression in prostate cancer tissues at 10x and 40x magnification. (**G**) Relative mRNA expression of the indicated genes upon Zoledronic acid (Zol) treatment. Data are plotted relative to the PBS control sample. N = 3; Error bars = SD. *$p < 0.05$; **$p < 0.01$; ***$p < .001$. (**H**) 22Rv1 cells were transfected with the reporter plasmid, where an endogenous ALDH1A1 promoter regulates luciferase expression. Transfected cells were treated with Zoledronic acid (Zol) at the indicated concentrations. An empty plasmid was used as a control. N = 3; Error bars = SD. *$p < 0.05$.

Supplementary Figure 4

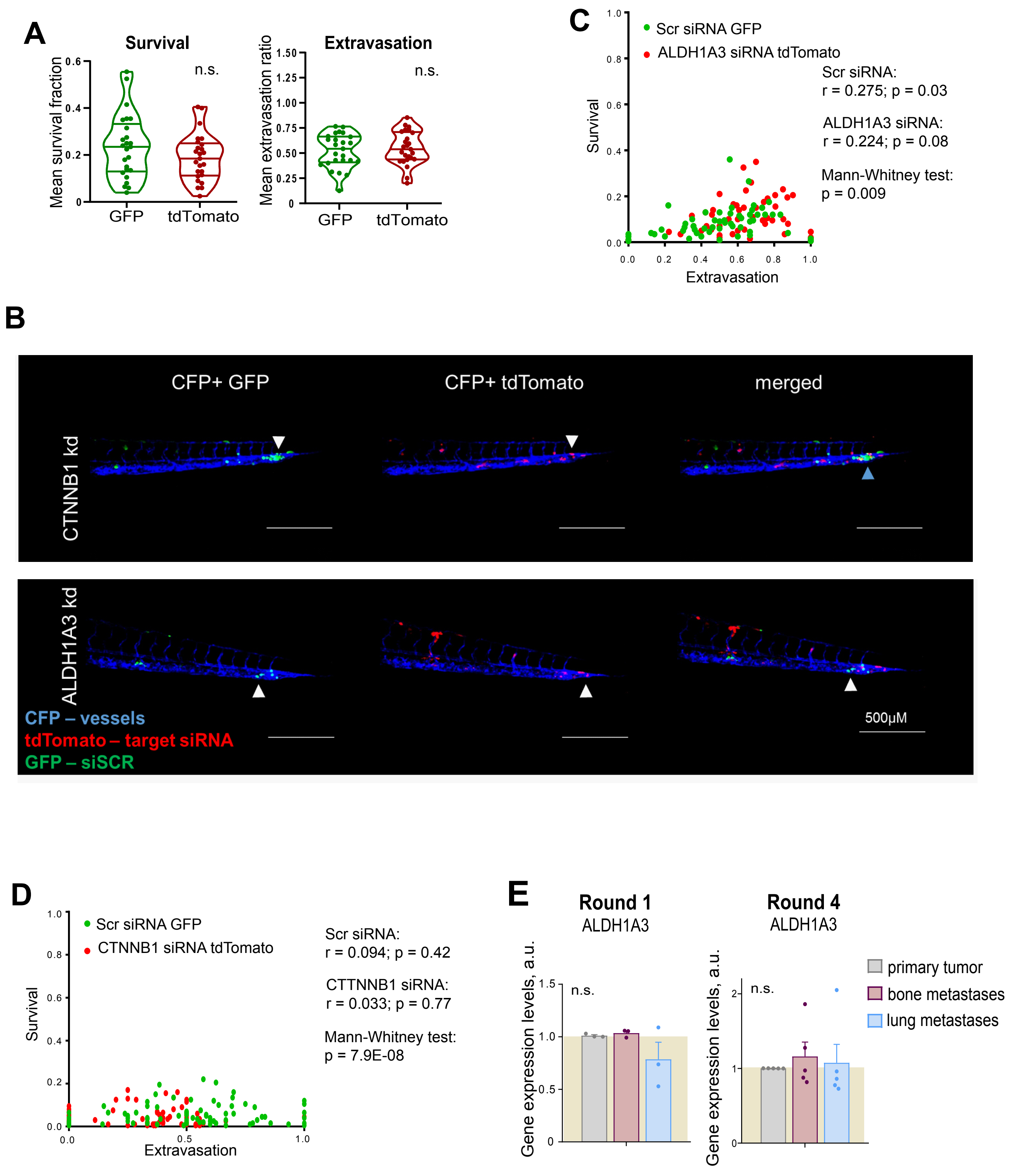

Supplementary Figure 4. Survival and extravasation potential of color-coded PC3 cells. (**A**) Survival in the bloodstream and extravasation potential of untreated color-coded PC3 cells. N = 25 (GFP); N = 25 (tdTomato). (**B**) Representative fluorescent images of the zebrafish tail in the CTNNB1 and ALDH1A3 samples. CFP – vessels; tdTomato – color-coded prostate cancer cells PC3 cells transfected with target siRNA; GFP – color-coded prostate cancer cells PC3 cells transfected with Scr siRNA. Scale bars = 500 μM. Arrows indicate extravasated cells. (**C**) Correlation of *in vivo* cell survival and extravasation in response to the scrambled (Scr) siRNA transfection (GFP positive cells) or ALDH1A3 siRNA transfection (tdTomato positive cells). Dissimilarity of cell survival and extravasation after Scr siRNA or ALDH1A3 siRNA transfection was evaluated by the data dimensionality reduction followed by the Mann-Whitney U test. (**D**) Correlation of *in vivo* cell survival and extravasation in response to the scrambled (Scr) siRNA transfection (GFP positive cells) or CTNNB1 siRNA transfection (tdTomato positive cells). (**E**) qPCR analysis of ALDH1A3 expression in the PC3 cells originating from different sites: primary tumors, bone marrow metastases, and lung metastases. Cells were passaged in mice in four rounds and the sublines from the 1st and 4th rounds were taken for the comparative analysis. The data is plotted relative to the primary tumor samples. N ≥ 3; Error bars = SEM. *p < 0.05.

Supplementary Figure 5

A

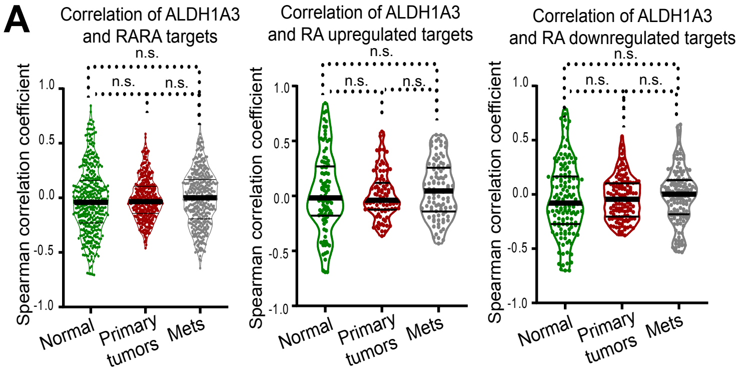

C

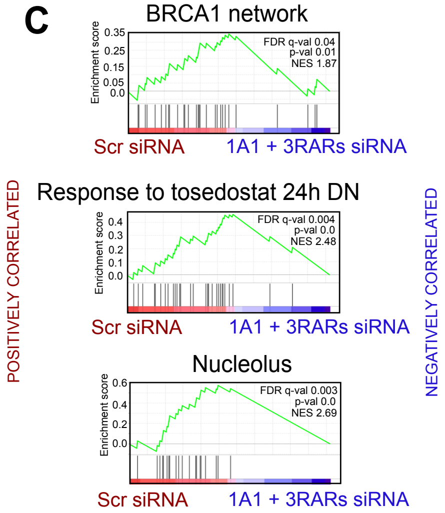

B

Genes overlapping after knockdown of ALDH1A1 and all 3x RARs (106 upregulated and 119 downregulated)

ABRAXAS1	AC005332.6	AC018521.5	AC069281.2	ACOT13
AKR7A2	AL022328.4	AL160006.1	AL354892.2	ALAD
ALDH1A3	ARHGEF37	BBS12	BICDL2	BMP8A
BRD3OS	BTD	C1QTNF1	C6orf62	C8orf37-AS1
CCDC74A	CFDP1	CHRD	COA5	COL1A1
CPE	CSAG3	CSNK1D	DOK4	DZANK1
ELN	ESCO1	ETNK2	FAM117B	FAM174B
FAM210B	FAM78A	FRA10AC1	FZD7	GDPD1
GPX3	GRHPR	GRN	GULP1	H3-3A
HDAC11	HELZ2	HMGCL	HOXA11- AS	HS1BP3
IGSF3	JARID2	KBTBD2	KIAA1958	KIF13B
KNDC1	LINC00853	LINGO3	LPIN2	LRCH4
LRRC6	LYN	LYRM9	MAGED1	MAP1LC3B
MAST3	MCC	MED9	MSN	NOA1
PADI2	PALM	PDZRN3	PHYH	PLEKHA5
PPP2R3A	PPP6C	PREP	PRKX	PSMB4
PTK7	RFFL	RNF24	RUFY1	SEMA6B
SEPTIN8	SLC25A42	SLC7A8	SMPD3	SORT1
SPG21	SPHK2	SPRY3	SSPOP	TCEAL1
TCTN2	TMEM107	TMEM14C	TPGS2	VANGL2
ZDHHC9	ZFYVE21	ZKSCAN1	ZNF143	ZNF264
ZNF703				

AC046185.2	AC073857.1	AKR7L	ALG2	ARL2BP
ATF3	B3GALNT2	B4GALT4	BATF3	BCL7B
C12orf43	C2CD3	CAMSAP1	CCDC137	CCN1
CCNJ	CDC42SE1	CENPBD1P1	CENPX	CRLF1
DCLRE1C	DERL2	DLEU1	DNAJB11	EEF1AKNMT
ELMOD3	ERICH1	FARSA	FBXO16	FUT4
GCC1	GEMIN5	GMPPB	GOLGA2P5	GPR180
GRK6	H2AC8	HSD17B7P2	HSPA5	HYOU1
KDM8	LIPT1	LLPH	LSM6	MARS2
MED8	METTL1	MLX	MPV17L2	MRPS12
MTRF1	MYO19	MYO1C	NFE2L1-DT	NOP16
NOP2	NOP56	NPM3	PDCD2	PGAM5
PINX1	PLK3 ←	PMAIP1	PNO1	POLR3A
POLR3D	POLR3E	PPIF	PREB	PRKAB1
PRR22	PRRG4	PTDSS1	PTMA	QTRT2
RAB8A	RAE1	RCL1	RGS16	RIOK1
RIOX2	RMC1	RPS19BP1	SARNP	SEC61A1
SLC16A6	SLC1A3	SLC20A1	SLC35E1	SLC5A6
SLC9A2	SMG9	SNHG15	STAG3L2	STIMATE
STOML1	TIMM22	TIMM44	TMEM138	TMEM184A
TMEM41A	TNFRSF10A	TNFRSF10A- AS1		TRAK1
TRAM2	TRMU	TRPM2	TTI1	TYMS
UCK1	UCK2	USP38	WDR77	XDH
ZBTB2	ZDHHC23	ZNF256	ZNF530	ZNF696

D

Up in ALDH1A1 siRNA/3xRAR siRNA – Down in ALDH1A3 siRNA

AC005332.6	KBTBD2
ALDH1A3	PDZRN3
BICDL2	PREP
CFDP1	PRKX
FAM117B	RFFL
FAM210B	RUFY1
FAM78A	SEPTIN8
FZD7	SLC7A8
HMGCL	TCTN2
HS1BP3	TMEM14C
JARID2	ZDHHC9

Down in ALDH1A1 siRNA/3xRAR siRNA – Up in ALDH1A3 siRNA

CCN1
CCNJ
FBXO16
MLX
NPM3
PLK3 ←
POLR3A
RGS16
RIOX2
SLC35E1
STAG3L2

F

Promoter ID: PLK3_1
Transcription factor motif: RARA
A cut-off (p-value) of 0.001

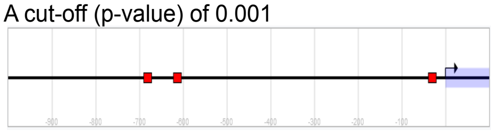

RARA [p-value = 0.001]: -690, -622, -39

Promoter ID: PLK3_1
Transcription factor motif: AR
A cut-off (p-value) of 0.01

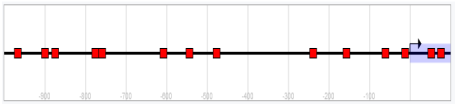

AR [p-value = 0.01]: -975, -908, -883, -784, -767, -616, -552, -485, -247, -165, -69, -21, 44, 68

E

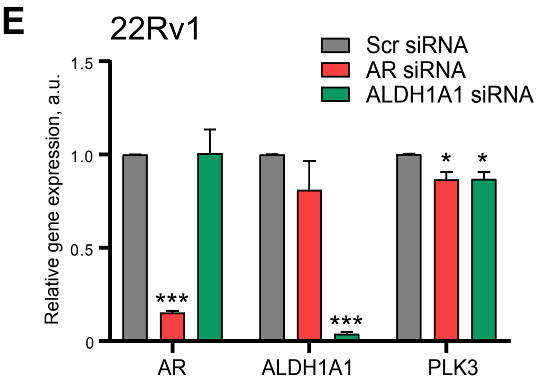

Supplementary Figure 5. Analysis of the ALDH1A1 and ALDH1A3-related transcriptional signatures. (**A**) Correlation of ALDH1A3 expression levels with the expression of the previously described RARA transcriptional targets [11] and genes reported to be up- or downregulated in response to RA treatment [11] in normal tissues (MSKCC dataset, n = 29), primary tumors (MSKCC dataset, n = 131), and metastatic tumors (MSKCC dataset, n = 19). Statistical analysis was performed by the Kruskal-Wallis rank sum test for multiple independent samples. Conover p-values were further adjusted by the Benjamini-Hochberg FDR method; n.s. – non-significant. (**B**) A list of genes overlapping after the knockdown of ALDH1A1 and all 3 retinoid transcriptional factors. (**C**) Gene Set Enrichment Analysis (GSEA) [12, 13] revealed that gene signature similarly deregulated by ALDH1A1 and retinoid receptors is associated with BRCA1 signaling, cell response to the anti-proliferative and anti-metastatic drug CHR-2797 (tosedostat) [14], and nucleolus functions; NES: normalized enrichment score. (**D**) Genes similarly regulated by ALDH1A1, RARs and RXRA knockdown, but oppositely regulated by ALDH1A3. (**E**) RT-qPCR analysis of PLK3, AR, and ALDH1A1 expression in 22Rv1 cells upon ALDH1A1 and AR knockdown. N=3; Error bars = SD; *$p < 0.05$; ***$p < 0.001$. (**F**) Analysis of the PLK3 gene promoter using The Eukaryotic Promoter Database (EPD) revealed putative RARA and AR binding elements.

Supplementary Figure 6

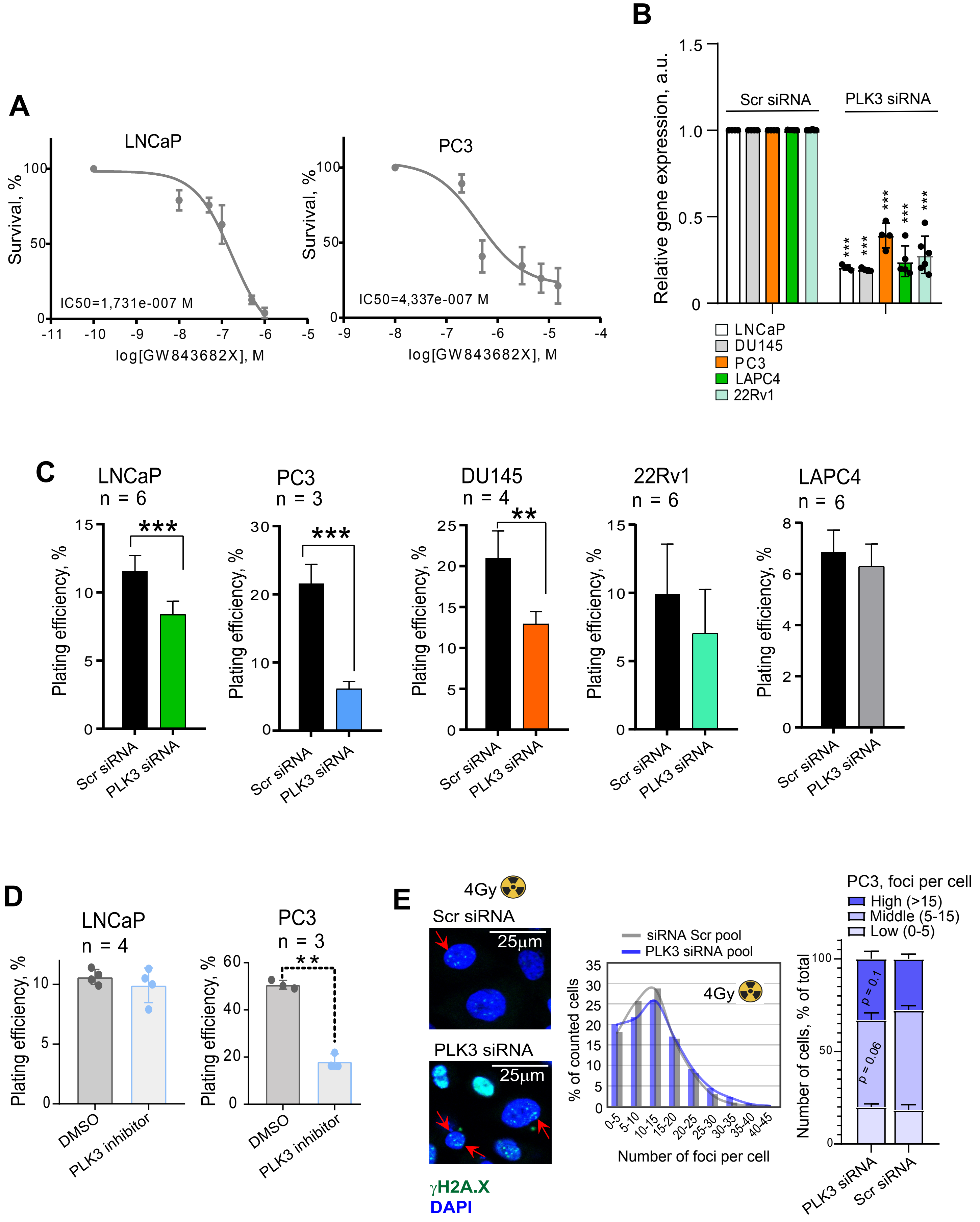

Supplementary Figure 6. Functional characterization of PLK3 in prostate cancer cell lines. (**A**) The CellTiter-Glo viability and proliferation analysis of LNCaP and PC3 cells in response to the treatment with PLK3 inhibitor GW843682X. IC_{50} values were determined after 48 h (n = 3) of treatment with the drug; N= 3; Error bars = SD. (**B**) RT-qPCR validation of the PLK3 knockdown in prostate cancer cells. Data are plotted relative to the Scr siRNA. N≥3; Error bars = SD; ***p<0.001. (**C**) Plating efficiency of prostate cancer cells after transfection with scrambled (Scr) siRNA or PLK3 siRNA. Error bars = SD; ***p<0.001; **p<0.01. (**D**) Plating efficiency of prostate cancer cells after 24 h pre-treatment with PLK3 inhibitor GW843682X at IC_{50} concentrations (for LNCaP cells, $IC_{50} = 1.73 \times 10^{-7}$ M; for PC3 cells, $IC_{50} = 4.34 \times 10^{-7}$ M). Cells treated with DMSO were used as control. N≥3; Error bars = SD; **p<0.01. (**E**). DNA double-stranded breaks (DSBs) were analyzed in PC3 by γ-H2A.X foci analysis in the individual cells 24 h after 4 Gy of X-ray irradiation. Scale bars = 25 µm. Arrows show the exemplary γ-H2A.X foci; the graphs show a distribution of cell nuclei by foci number after 4Gy of X-ray irradiation.

Supplementary references:

1. Peterziel H, Mink S, Schonert A, Becker M, Klocker H, Cato AC. Rapid signalling by androgen receptor in prostate cancer cells. Oncogene. 1999; 18: 6322-9.
2. Cojoc M, Peitzsch C, Kurth I, Trautmann F, Kunz-Schughart LA, Telegeev GD, et al. Aldehyde Dehydrogenase Is Regulated by beta-Catenin/TCF and Promotes Radioresistance in Prostate Cancer Progenitor Cells. Cancer Res. 2015; 75: 1482-94.
3. Mukha A, Kahya U, Linge A, Chen O, Lock S, Lukiyanchuk V, et al. GLS-driven glutamine catabolism contributes to prostate cancer radiosensitivity by regulating the redox state, stemness and ATG5-mediated autophagy. Theranostics. 2021; 11: 7844-68.
4. Power CA, Pwint H, Chan J, Cho J, Yu Y, Walsh W, et al. A novel model of bone-metastatic prostate cancer in immunocompetent mice. Prostate. 2009; 69: 1613-23.
5. Zolfaghari R, Mattie FJ, Wei CH, Chisholm DR, Whiting A, Ross AC. CYP26A1 gene promoter is a useful tool for reporting RAR-mediated retinoid activity. Anal Biochem. 2019; 577: 98-109.
6. Hess I, Boehm T. Intravital imaging of thymopoiesis reveals dynamic lympho-epithelial interactions. Immunity. 2012; 36: 298-309.
7. Peitzsch C, Cojoc M, Hein L, Kurth I, Mabert K, Trautmann F, et al. An Epigenetic Reprogramming Strategy to Resensitize Radioresistant Prostate Cancer Cells. Cancer Res. 2016; 76: 2637-51.
8. Hoffmann B, Lange T, Labitzky V, Riecken K, Wree A, Schumacher U, et al. The initial engraftment of tumor cells is critical for the future growth pattern: a mathematical study based on simulations and animal experiments. BMC Cancer. 2020; 20: 524.
9. Robinson D, Van Allen EM, Wu YM, Schultz N, Lonigro RJ, Mosquera JM, et al. Integrative clinical genomics of advanced prostate cancer. Cell. 2015; 161: 1215-28.
10. Salberg UB, Skingen VE, Fjeldbo CS, Hompland T, Ragnum HB, Vlatkovic L, et al. A prognostic hypoxia gene signature with low heterogeneity within the dominant tumour lesion in prostate cancer patients. Br J Cancer. 2022; 127: 321-8.
11. Hua S, Kittler R, White KP. Genomic antagonism between retinoic acid and estrogen signaling in breast cancer. Cell. 2009; 137: 1259-71.
12. Mootha VK, Lindgren CM, Eriksson KF, Subramanian A, Sihag S, Lehar J, et al. PGC-1alpha-responsive genes involved in oxidative phosphorylation are coordinately downregulated in human diabetes. Nat Genet. 2003; 34: 267-73.
13. Subramanian A, Tamayo P, Mootha VK, Mukherjee S, Ebert BL, Gillette MA, et al. Gene set enrichment analysis: a knowledge-based approach for interpreting genome-wide expression profiles. Proc Natl Acad Sci U S A. 2005; 102: 15545-50.
14. van Herpen CM, Eskens FA, de Jonge M, Desar I, Hooftman L, Bone EA, et al. A Phase Ib dose-escalation study to evaluate safety and tolerability of the addition of the aminopeptidase inhibitor tosedostat (CHR-2797) to paclitaxel in patients with advanced solid tumours. Br J Cancer. 2010; 103: 1362-8.
